# Metabolism of Epoxidised Withanolides by a Generalist and a Specialist Insect Species

**DOI:** 10.1002/open.202500359

**Published:** 2025-09-11

**Authors:** Marie Pauline Sell, Christian Paetz, Felix Feistel, David G. Heckel, Yannick Pauchet

**Affiliations:** ^1^ Institute of Cell and Systems Biology of Animals, Molecular Evolutionary Biology University of Hamburg 20148 Hamburg Germany; ^2^ Department of Insect Symbiosis Max Planck Institute for Chemical Ecology 07745 Jena Germany; ^3^ NMR/Biosynthesis Group Max Planck Institute for Chemical Ecology 07745 Jena Germany; ^4^ Department of Biochemistry Max Planck Institute for Chemical Ecology 07745 Jena Germany; ^5^ Emeritus Group Entomology Max Planck Institute for Chemical Ecology 07745 Jena Germany

**Keywords:** *Chloridea*, *Heliothis*, *Physalis*, *subflexa*, *virescens*, withanolides

## Abstract

Major secondary metabolites of *Physalis* plants, epoxidized withanolides, have a potent feeding deterrent and growth inhibitory effect on most herbivorous insects. Caterpillars of the specialist moth species *Heliothis (Chloridea) subflexa* consume only *Physalis* fruits, whereas the closely related generalist *Heliothis (Chloridea) virescens* feeds on 14 different plant families, but not *Physalis*. The two species have different physiological responses to dietary withanolides, so it is wondered whether they metabolize withanolides differently. The *Physalis peruviana* plants are grown in a [^13^C]CO_2_‐supplied atmosphere, 4*β*‐hydroxywithanolide E is isolated and purified from the leaves, and the compound is fed to the caterpillars. Subsequent high‐performance liquid chromatography with diode array UV‐vis detection coupled to high‐resolution electrospray ionization mass spectrometry (HPLC‐DAD‐HRESIMS) and nuclear magnetic resonance (NMR) analyses of the main metabolite isolated from the frass show that both species convert 4*β*‐hydroxywithanolide E mainly to withanolide S, probably by the action of an epoxide hydrolase. Withanolide S is completely characterized regarding its NMR and electronic circular dichroism data. To date, this is the first study to analyze the fate of withanolides after ingestion by insects.

## Graphical abstract

Larvae of *Heliothis (Chloridea) virescence* and *subflexa* have the ability to convert the epoxidized terpenoid 4β‐hydroxywithanolide E (4BHWE) to withanolide S. The conversion is found by feeding the larvae an artificial diet supplemented with ^13^C‐labeled 4BHWE. The metabolite is recovered from the larval frass and characterized by high performance liquid chromatography coupled to high resolution electrospray ionization mass spectrometry (HPLC‐HRESIMS), nuclear magnetic resonance (NMR), and electronic circular dichroism (ECD) spectroscopy.

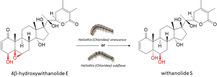

## 1 Introduction

*Physalis peruviana* or Cape gooseberry (Solanaceae) is a flowering plant native to South America that produces edible fruits. The plant produces a variety of terpenoid defense compounds, withanolides, and since their first detection in *P. peruviana*, many studies have reported the discovery of novel withanolides and derivative forms.^[^ ^1^, ^2^, ^3^, ^4^ ^–^ ^5^ ^]^ Although the exact ecological role and the mode of action are not fully understood, withanolides are presumed to act as a defense to prevent herbivorous attack.^[^ ^6^ ^]^ Feeding on *Physalis spp.* thus requires the ability to tolerate and/or to metabolize withanolides, as well as acylsugars, which are also produced by *Physalis*.^[^ ^7^ ^]^ *Physalis* fruits are covered by a calyx, a hollow balloon‐like structure that completely encloses the fruit. *Chloridea (Heliothis) subflexa* caterpillars, which are specialist herbivores on *Physalis*, spend their entire larval phase within the calyx, feeding on the fruit.^[^ ^8^ ^,^ ^9^ ^]^ The closest relative of *subflexa* is *Chloridea (Heliothis) virescens,* a generalist herbivore that feeds on 14 different plant families, including many agricultural crops. It shares an overlapping habitat in North and South America, but has not been observed feeding on *Physalis* plants in the field.^[^ ^10^ ^]^ The two species respond differently to withanolides added to the artificial diet: *subflexa* growth increases while *virescens* growth decreases.^[^ ^11^ ^]^ Withanolides stimulate the expression of immune genes in *subflexa*, but inhibit immune gene expression in *virescens*.^[^ ^11^ ^]^

The reason for the adverse effect of withanolides on insects is not fully understood. Some withanolides contain epoxide groups that are active against nucleophiles.^[^ ^12^ ^,^ ^13^ ^]^ A general reactivity toward biomolecules can therefore be assumed. In a *Drosophila* cell line expressing the ecdysone receptor, several withanolides were found to be ecdysone antagonists at low concentrations and to be cytotoxic at high concentrations.^[^ ^14^ ^]^ Therefore, it has been suggested that withanolides may interfere with ecdysone signaling, but a direct phytoecdysteroid function of withanolides has not yet been confirmed. Another possible target of withanolides could be cholinesterases, since withanolides have been shown to inhibit vertebrate acetylcholinesterase and butyrylcholinesterase.^[^ ^15^ ^,^ ^16^ ^]^

The fate of withanolides after uptake in vivo has so far only been studied in mammals. In rats, the major metabolic pathways for physalin A were sulfonation, reduction, and hydroxylation.^[^ ^17^ ^,^ ^18^ ^]^ The metabolites of withaferin A after incubation with rat liver microsomes have been analyzed using HPLC‐MS. The authors observed rapid elimination and discovered seven metabolites as a result of putative hydrogenation, hydroxylation, and hydrolysis reactions, but none of the structures of the metabolites could be elucidated.^[^ ^19^ ^]^ We are not aware of any studies on the metabolism of withanolides by insects.

**Figure 1:**
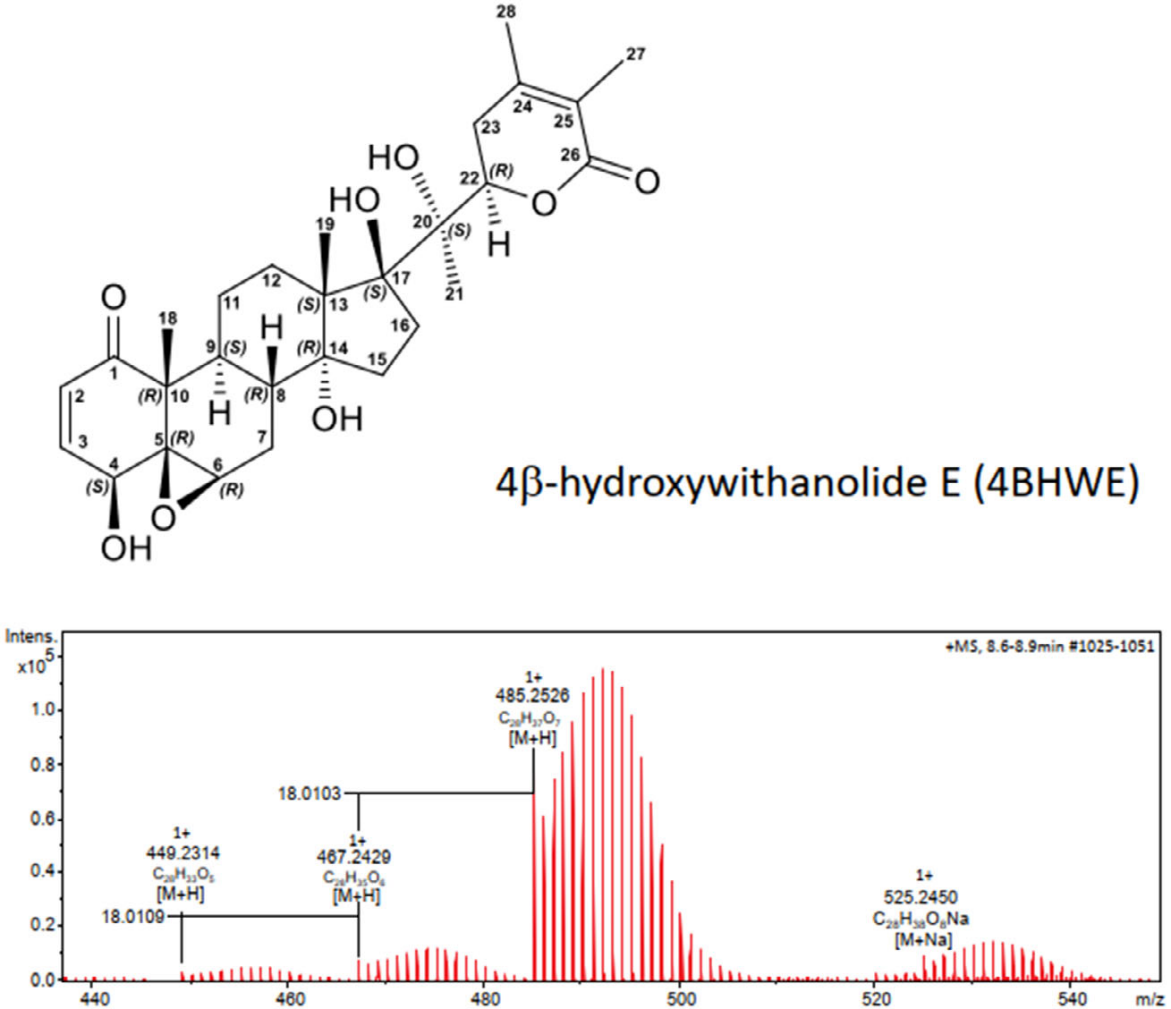
Structure and high resolution mass spectrum (ESI, pos. ionization) of 4*β*‐hydroxywithanolide E (4BHWE). The lower part of the figure shows the characteristic mass spectrum of the biogenically ^13^C‐labeled compound derived from a [^13^C]CO_2_ incubation experiment with *P. peruviana*. Each distribution of isotopomers is preceded by a pronounced ^12^C signal from which the molecular composition can be calculated. Using such labeled compounds as probes in metabolism studies facilitates the identification of metabolites. For a detailed analysis of the mass spectra and the fragmentation, see the Supporting Information.

Therefore, we aimed to investigate how insects metabolize withanolides and whether there are differences between *virescens* and *subflexa*, given their different responses to dietary withanolides. We cultivated *P. peruviana* plants in the greenhouse and isotopically labeled plant metabolites with [^13^C]CO_2_ using a previously described method.^[^ ^20^ ^]^ We then isolated and purified ^13^C‐labeled 4*β*‐hydroxywithanolide E (hereafter referred to as 4BHWE) and fed it to larvae of both species in an artificial diet. The ^13^C‐labeled 4BHWE displays a distinctive signature in its mass spectra, enabling the straightforward identification of its metabolic products (**Figure** **1**). The frass was then analyzed by high‐performance liquid chromatography with diode array UV‐Vis detection coupled to high‐resolution electrospray ionization mass spectrometry (HPLC‐DAD‐HRESIMS), and nuclear magnetic resonance (NMR) spectroscopy to identify the ^13^C‐labeled withanolide metabolites.

## 2 Experimental Section

### 2.1 Insect Rearing

*Heliothis* is a polyphyletic genus, and *virescens* and *subflexa* were recently reassigned to the monotypic genus *Chloridea*,^[^ ^21^ ^]^ but since there is an extensive literature on heliothine plant‐insect interactions, we will use the previous genus name for continuity. Larvae were originally collected in Florida (*subflexa*) and North Carolina (*virescens*) and reared at North Carolina State University until their transfer to the Max Planck Institute for Chemical Ecology in Jena, Germany (MPI‐CE), in 2005 and 2006, respectively. All larvae were grown on a General Purpose Lepidoptera (GPL) artificial diet based on wheat germ and soy flour. The diet was prepared by adding 19 g agar to 144 g Dry Mix F9772 (Frontier Scientific Services, Newark, DE, USA) per 875 mL of distilled water. After pupation, individual larvae were kept in single 20 mL plastic cups (Ultra Clear PET Soufflé Portion; Dart Container Corporation, Mason, MI, USA) until they eclosed. Pupae and mating couples were kept in a climate chamber (Snijders Produktie B.V., Tilburg, The Netherlands) at 26 °C, 55 ± 10% relative humidity, and 16:8 h light/dark cycle (L:D). Emerged adults were kept at 12 °C 55 ± 10% relative humidity, and 16:8 h (L:D) and fed with a 10% honey water solution (v/v). Single pair mating was performed for balanced breeding. All mating couples were set up in plastic coffee cups covered with egg cloths made of gauze. Egg cloths with fertilized eggs were collected and transferred to Petri dishes containing the GPL diet.

### 2.2 Plant Rearing

*P. peruviana* was grown in the greenhouse of the MPI‐CE. Cuttings were grown under a 14:10 h (L:D) light regime and a temperature range of 18–21 °C (night) and 21–23 °C (day). The average humidity was set at 50–60%. Older plants were kept in a chamber with 16:8 h (L:D) at 19–23 °C (night) and 23–25 °C (day) with a humidity range of 45–55%. All plants were grown on a (70/200 v/v) soil mixture of Futterpflanzensubstrat/Tonsubstrat TS1 (Klasmann‐Deilmann, Geeste, Germany).

### 2.3 Isotopic Labeling of *P. peruviana* Plants

^13^C‐isotope labeling using a controlled ^13^CO_2_ atmosphere was performed in a growth chamber as previously described.^[^ ^20^ ^]^ Three *P. peruviana* plants were pruned before being transferred to the chamber. The plants were grown using the same light and temperature settings as those described for greenhouse cultivation. At the start of each light period, [^13^C]CO_2_ (600 ppm) was pulse‐injected into the chamber via a mass flow controller (Red‐Y Smart Controller GSC, Vögtlin, Muttenz, Switzerland). This concentration was kept constant during light periods. During dark periods, the CO_2_ produced by respiration was removed by pumping the chamber atmosphere through a sodium hydroxide scrubber cartridge. The labeling experiment was performed in late autumn, after the normal growth period, and therefore, we observed a very slow growth rate with minimal ^13^CO_2_ consumption. After 75 days, the plants were removed and the newly grown leaves were harvested and snap frozen in liquid N_2_.

### 2.4 Extraction and Isolation of 4BHWE from ^13^C‐Labeleled Plant Material

The plant material obtained from ^13^CO_2_‐labeling of *P. peruviana* was lyophilized and ground into powder in a laboratory mill (IKA M20 universal mill, IKA‐Werke GmbH & Co. KG, Staufen, Germany). The powder (5.5 g) was then transferred to a 500 mL Erlenmeyer flask. Five extractions were performed under constant shaking using a total volume of 1000 mL MeOH. The combined methanolic extracts were filtered through a folded MN paper filter (Macherey‐Nagel [MN] GmbH & Co. KG, Düren, Germany), and the solvent was evaporated under reduced pressure using a rotary evaporator. The residue was then resuspended in 200 mL Milli‐Q H_2_O and extracted 3 times with a total of 300 mL n‐hexane using a separating funnel. The aqueous suspension was then loaded onto a conditioned and equilibrated MCI GEL CP20P polymer resin column and separated by medium‐pressure liquid chromatography (MPLC). An aliquot of each fraction was analyzed by HPLC‐DAD‐HRESIMS. We found 4BHWE to be the main withanolide of the extract. The fractions containing 4BHWE were pooled and re‐purified by MPLC on a C‐18 column. After the fractions were analyzed by HPLC‐DAD‐HRESIMS and selected regarding their 4BHWE content, the final purification was accomplished by semi‐preparative HPLC using an MN reverse phase column (phenyl‐hexyl modification). The fractions containing pure 4BHWE were pooled, and the solvent was removed using N_2_ gas. A total amount of 1.5 mg of 4BHWE was obtained (Figure 1). For chromatographic details, see the mass spectrometry and chromatography chapter and the Supporting Information for analytical data.

### 2.5 Feeding Assays Using 4BHWE

Four individual fourth‐instar larvae per species were fed 4BHWE. For feeding assays, 1 mL of GPL diet was pipetted into each well of a 24‐well plate and allowed to solidify. The diet was then surface sterilized under UV light for 15 min. Isolated 4BHWE (1.5 mg) was suspended in 1 mL 60% MeOH, and 125 µL was transferred to the diet of each well and evaporated for 4 h. Each supplied well contained ≈190 µg of the ^13^C‐labeled compound. Larvae were allowed to feed on this diet for 48 h. Frass was pooled per species, then lyophilized and subjected to analysis.

### 2.6 Mass Spectrometry and Chromatography

HPLC‐DAD‐HRESIMS analyses were performed on an Agilent 1260 HPLC system, consisting of a combined degasser/quaternarypump G1311B, an autosampler G1367E, a column oven G1316A, and a photodiode array detector G1315D (Agilent Technologies GmbH, Waldbronn, Germany) connected to a Bruker Compact OTOF mass spectrometer (Bruker Daltonics GmbH, Bremen, Germany). Mass spectra were obtained using electro spray ionization (ESI) in either positive or negative ionization mode using a mass range of *m/z* 50 to *m/z* 1300. Standard parameters for small molecule analysis were used as implemented in Bruker Compass ver.1.9. Chromatographic parameters for HPLC‐DAD‐HRESIMS analyses were as follows: Column, Agilent InfinityLab Poroshell 120 EC‐C18, 50 x 4.6 mm, 2.7 µm pore size, flow rate 0.4 mLmin^−1^, solvent A, H_2_O, solvent B, MeCN, both containing 0.1% formic acid. Gradient settings: initial, 5% B for 1 min, then to 95% B at 9 min, kept isocratic for 2 min, and then back to 5% B at 12 min. A 5 min pre‐run with the initial conditions preceded each injection. The column oven temperature was set to 40 °C.

A two‐step MPLC (MPLC, Biotage Isolera One, Biotage Sweden AB, Uppsala, Sweden) was used for the coarse purification of 4BHWE. At first, a column was prepared using MCI gel (CP20P, Merck KGaA, Darmstadt, Germany). The gel was suspended in MeOH and filled into an empty MN MPLC column (20 × 5 cm) equipped with a sintered PE bottom filter. After the gel had settled, the top filter was installed and the column was locked. Medium‐pressure chromatographic conditions were as follows: Flow rate, 100 mLmin^−1^. Solvent A: water containing 0.2% formic acid, solvent B: MeCN. Using the MPLC settings for Biotage SNAP Ultra C18 400 g columns, the column was purged with another 400 mL B and then equilibrated with 800 mL A. Next, the aqueous de‐fatted *P. peruviana* leaf extract was loaded into the column. For separation, a linear gradient was employed, starting from 0% B to 100% B in 33 column volumes (CV). Each fraction had a volume of 45 mL. The fractionation was monitored by UV detection at 230 nm and 250 nm. UV‐active constituents eluted from fraction 17–33. Starting at fraction 38, the column was flushed with 1500 mL of acetone. 4BHWE‐containing fractions were pooled and underwent a second MPLC separation. For this, a Biotage SNAP Ultra C‐18 12 g column was used, and the flow rate was 20 mLmin^−1^. The mixture, dissolved in 2 mL MeOH, was loaded into the equilibrated column (20% B) and gradient elution was applied. Starting with 20% B for 1 CV, the gradient reached 80% B after 11 CV, which was kept isocratic for 4 CV. The column was finally purged with 100% B for one CV. After HPLC‐DAD‐HRESIMS analysis, the 4BHWE‐containing fractions were subjected to a final HPLC purification.

HPLC separations (for purification of 4BHWE and 4BHWE metabolites from frass) were accomplished using an Agilent 1100 HPLC system, consisting of a degasser G1322A, a binary pump G1312A, an autosampler G1313A, and a photodiode array detector G1315B. The column outlet was connected to an Advantec CHF122 SB fraction collector (Advantec Toyo Kaisha Ltd., Tokyo, Japan) triggered by the HPLC with an Agilent relay contact board. Chromatographic conditions: MN phenyl‐hexyl column (Macherey‐Nagel GmbH & Co. KG, Düren, Germany), 250 × 4.6 mm, 5 µm pore size, solvent A, H_2_O, solvent B, MeCN, both with 0.1% formic acid, the flow rate was 1 mL min^−1^. Gradient: initial 5% B for 5 min, 40% B at 30 min, 100% B at 32 min, kept isocratic until 42 min, 5% at 45 min, kept isocratic until 50 min. The column oven temperature was set to 35 °C.

### 2.7 Extraction of ^13^C‐Labeled Metabolites from Frass

The freeze‐dried frass samples were extracted 3 times by shaking at 5000 rpm for 30 s with MeOH (5 mL) in a Bertin Minilys cell disruptor (Bertin Technologies SAS, Montigny‐le‐Bretonneux, France) using 7 mL vessels containing 1.4 mm ZrO_2_ beads. After each extraction step, the vessel was centrifuged at 6000 rpm for 2 min, and the supernatant was removed with a pipette. The pooled extracts were then transferred to a 15 mL Falcon tube and again centrifuged at 15.000 rpm for 5 min. The supernatant was then filtered through a 20 mm o.d. 0.22 µm PTFE disc filter, and an aliquot was analyzed by HPLC‐DAD‐HRESIMS. The chromatograms obtained were manually examined for signatures of ^13^C‐labeled metabolites. We isolated 150 µg of the main 4BHWE metabolite by HPLC.

### 2.8 NMR Spectroscopy

NMR spectra were recorded on a Bruker Avance III HD 700 MHz spectrometer, equipped with a cryoplatform and a 1.7 mm TCI microcryoprobe, or on a Bruker Avance III HD 500 MHz NMR spectrometer equipped with a cryoplatform and a 5 mm TCI cryoprobe (Bruker Biospin GmbH, Rheinstetten, Germany). All spectra were recorded at 298 K with MeOH‐*d*_3_ as solvent. Chemical shifts were referenced to the residual solvent peaks at *δ*_H_ 3.31 and *δ*_C_ 49.15. Data acquisition/analysis was performed using the standard pulse programs/processing routines implemented in Bruker TopSpin ver.3.6.1.

### 2.9 Electronic Circular Dichroism (ECD) Spectroscopy

Chiroptical measurements were carried out using a Jasco J‐810 instrument (Jasco Deutschland GmbH, Pfungstadt, Germany) equipped with a 1 mm path length quartz cuvette (350 µl volume). Methanol was used as a solvent. Instrument control and data acquisition were accomplished using the Jasco SpectraManager II software.

### 2.10 Molecular Modelling

Structural formulae were generated using the Revvity ChemOffice suite version 23.1.2.7. 2D structures created in ChemDraw were optimized using the MM2 tool in Chem3D and imported into Gaussian 16 (Gaussian Inc., Wallingford, CT, USA)^[^ ^22^ ^]^ as SYBYL2 type (.mol2) files. A semi‐empirical optimization (PM6) was performed in Gaussian 16, and the resulting structures underwent conformational analysis in GMMX (implemented in GaussView). The conformer structures were used for DFT calculations (first ground state, then with the TDA‐SCF method using the B3LYP‐6‐311G(2d,p) basis set; fifty frequencies using the default solvation settings for methanol were calculated for the ECD curve). For data visualization and calculation setup GaussView ver.6.1.1 (Semichem Inc., Shawnee Mission, KS, USA)^[^ ^23^ ^]^ was used.

## 3 Results and Discussion

### 3.1 Isolation of 4BHWE for Feeding Assays

The key element in our assay was a previously identified^[^ ^24^ ^]^ naturally biosynthesised probe, 4BHWE, which we obtained by isolation from ^13^C‐labeled *P. peruviana* plant material (Figure S1 and S2, Supporting Information). The labeling was achieved by growing plants in a [^13^C]CO_2_ atmosphere under controlled conditions. The degree of ^13^C‐labeling at each position of the molecule depends on its biosynthesis, and therefore a characteristic isotopologue pattern occurs in the mass spectra. Our method of ^13^C‐labeling yields constituents with a typical bell‐shaped isotopologue pattern that is preceded by a larger ^12^C mass peak (from the product biosynthesised prior to [^13^C]CO_2_ incubation), which allows the calculation of the metabolite's molecular composition (Figure 1 and Figure S3–S17, Supporting Information). Metabolites arising from biochemical transformations of such labeled chemical probes also have a unique labeling pattern and can hence be identified easily.

### 3.2 Mass Spectrometric Analysis of Frass after Feeding 4BHWE

**Figure 2:**
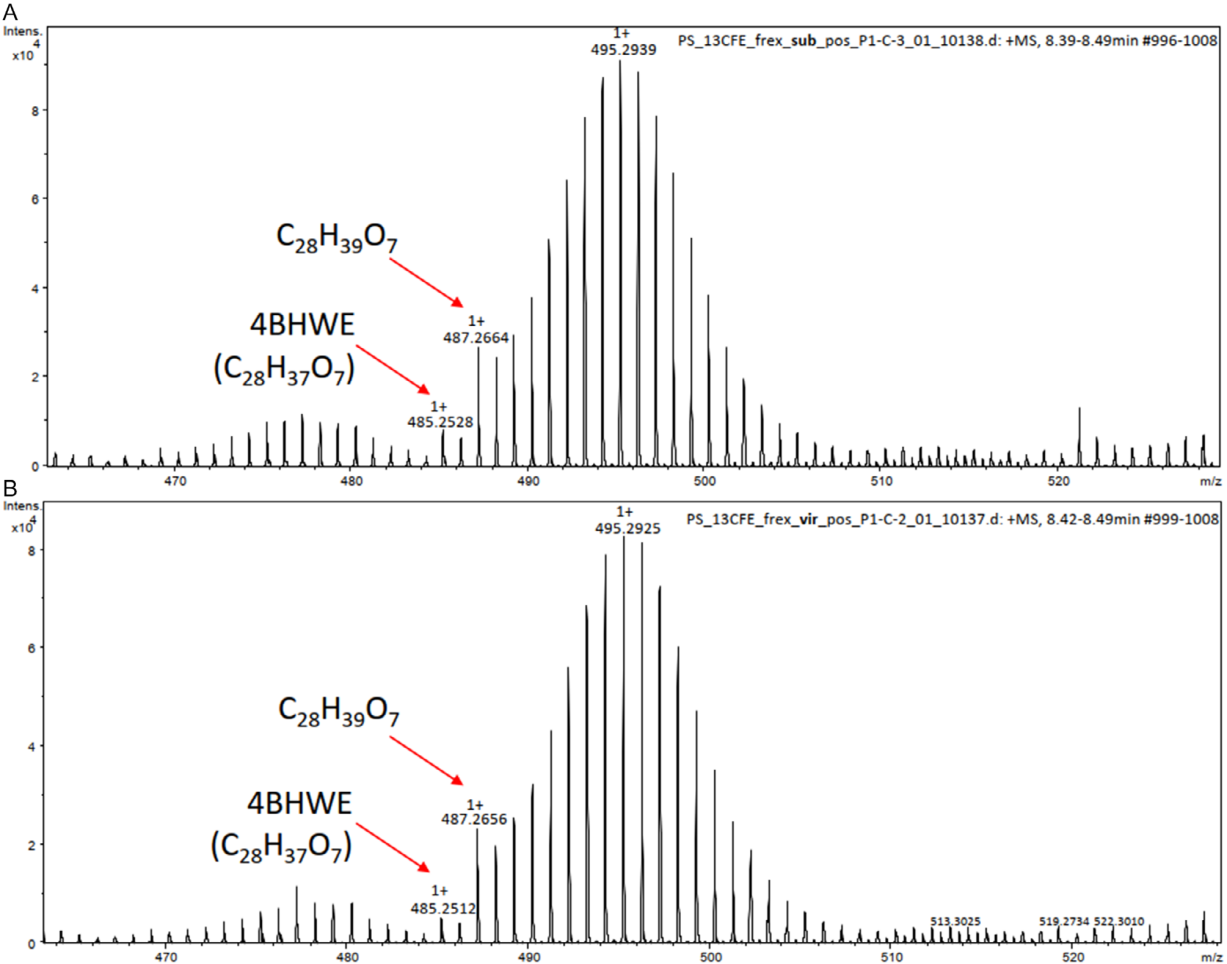
Positive mode HRESIMS spectra of the main metabolite after feeding 4BHWE to *C. (H.) subflexa* (A) and *C. (H.) virescens* (B). Due to partial coelution of probe and metabolite, the mass spectra of both compounds are mixed.

After feeding 4BHWE to the larvae, we collected their frass and examined the methanolic extracts for isotopologue patterns. We observed that the basic withanolide structure was preserved in the metabolites, as shown in the extracted ion chromatograms, where a fragment of m/z 169.0859 [M + H]^+^ indicates the cleavage of the unsaturated lactone moiety from the withanolide (Figure S18, Supporting Information). Additionally, it was found that the metabolites eluted in the same retention range in both species. A metabolite with the sum formula C_28_H_40_O_8_ (*m/z* 549.2691, calcd. from M + HCOOH‐H, *m/z* 549.2705, C_29_H_41_O_10_, neg. mode ESIMS, and *m/z* 487.2670, calcd. from M‐H_2_O+H, *m/z* 487.2690, C_28_H_39_O_7_, pos. mode ESIMS) was the major transformant in both species (**Figure** **2** and Figure S18, Supporting Information), which was partly overlapping with unmetabolised 4BHWE. We therefore conclude that 4BHWE is not metabolized differently in the two species. Compared to the 4BHWE probe, the molecular composition of the main metabolite consists of two additional hydrogens and can therefore be considered as a reduced form of the probe.

To obtain enough material for NMR structure elucidation, we combined the frass extracts of both species and separated this extract by HPLC (Figures S19 and S20, Supporting Information). The structure of the main metabolite was further elucidated by NMR.

### 3.3 Identification of the Main 4BHWE Derived Metabolite by NMR

**Figure 3:**
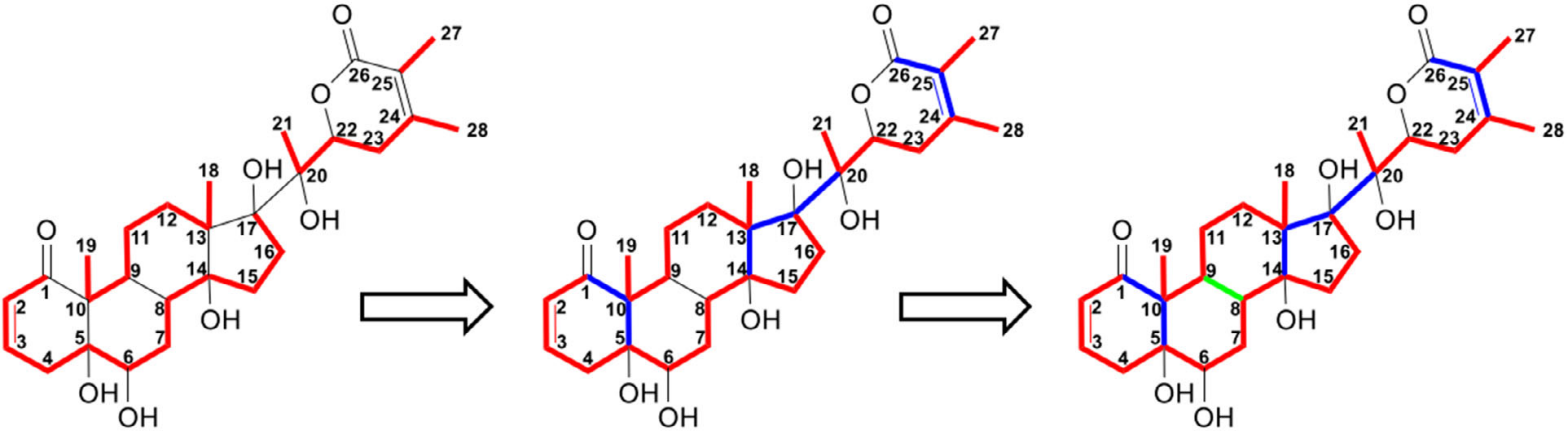
NMR elucidation workflow of the main 4BHWE metabolite. The left structure shows the fragments established from 1,1‐ADEQUATE data, indicated in red. The remaining junctions highlighted in blue were established from the long‐range ^1^H‐^13^C correlations of methyl functions in the HMBC spectrum. The identification of the six‐membered spin system of positions 6 to 12 by HSQC‐TOCSY data established the junction between positions 8 and 9 (highlighted in green in the structure on the right). Oxygenations were deduced from chemical shifts.

After isolating the main metabolite (C_28_H_40_O_8_) by HPLC, we analyzed its basic structure by NMR, interpreting data from ^1^H‐^13^C HSQC, HMBC, HSQC‐TOCSY, and 1,1‐ADEQUATE spectra. Unlike the usual NMR structure elucidation workflow, we did not make use of extensive interpretations of ^1^H NMR data. Instead, thanks to the high degree of ^13^C‐labeling in the metabolite, we established the structure by ^13^C‐^13^C correlations (1,1‐ADEQUATE). Also, the sensitivity of the other inverse ^1^H‐^13^C NMR experiments was greatly improved, which resulted in a straightforward workflow (Figure S21–S31, Supporting Information). We found a major change in ring A of the withanolide structure, where C‐4 lost its hydroxylation. The introduction of two hydrogens was therefore due to the opening of the *β*−5,6 epoxide. Given the established chemical composition and the oxygenation pattern, three already described structures for the metabolite were possible, namely withanolide S (CASRN 63,139‐16−2), physaperuvin G^[^ ^25^ ^]^ (CASRN 1,946,775‐11−6), and 4‐deoxywithaperuvin^[^ ^26^ ^]^ (CASRN 484,016‐15−1) (**Figure** **3** and Figure S40, Supporting Information).^[^ ^27^ ^,^ ^28^ ^]^

**Figure 4:**
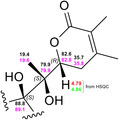
Comparison of the chemical shifts of the side chain (C17‐C20/C21‐C22) of 4BHWE (*δ*_C_ black and *δ*_H_ red) and withanolide S (*δ*_C_ magenta and *δ*_H_ green). The ^1^H chemical shifts of H22 have been extracted from HSQC data.

**Figure 5:**
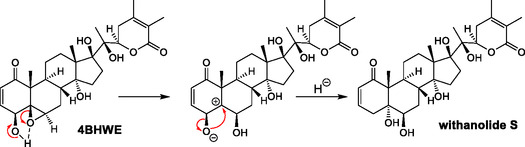
Hypothetical mechanism explaining the transformation of 4BHWE into withanolide S. The second step of the transformation allows in principle a nucleophilic attack on both sides of the carbonium atom in position 5, however, only one stereoisomer could be found. Such rearrangement is very unlikely to happen without enzymatic catalysis.

We then attempted to establish the stereochemistry of the metabolite by comparison with reported NMR chemical shift data and correlations in the ^1^H‐^1^H ROESY spectra (Figure S32 and S33, Supporting Information). The side chain stereochemistry 17(*S*),20(*S*),22(*R*) appears to be conserved, as shown by the very similar chemical shift values of 4BHWE and its metabolite (**Figure** **4**),^[^ ^29^ ^]^ and we also assumed that the epoxide transformation resulted in a 6*β*‐hydroxylated molecule (**Figure** **5**).

Physaperuvin G, described as a new compound and the 20(*R*) isomer of withanolide S,^[^ ^25^ ^]^ has NMR data very similar to that of our metabolite (Table S1, Supporting Information). The authors established the structure of this compound from an X‐ray analysis; however, they incorrectly interpreted the structure, considering differences in the NMR shift data obtained from measurements in different solvents. This method of stereochemical determination, which has previously been applied to a variety of withanolide structures, has been shown to be flawed.^[^ ^30^ ^]^ We did molecular modeling using the side chain geometry of 4BHWE and a 5*α*, 6*β*‐*trans*‐dihydroxylation, i.e., withanolide S, which yielded a structure identical to the X‐ray result of physaperuvin G (Figure S38, Supporting Information). We therefore conclude that the compound described as physaperuvin G^[^ ^25^ ^]^ is not a new compound and in fact is the same as withanolide S.

However, we could not determine the configuration at C‐5 of our metabolite with certainty using ROESY correlations alone (Figure S34 and S35, Supporting Information). Therefore, 4‐deoxywithaperuvin, reported to have the same side chain configuration as 4BHWE but with a 5*β*, 6*α*‐dihydroxylation, was also a possible candidate for the metabolite structure (Figure S40, Supporting Information). Whether 4‐deoxywithaperuvin or withanolide S (5*α*, 6*β*‐diOH) was present could be shown by ECD measurement (Figure S36 and S37, Supporting Information). The experimental ECD is in full agreement with the calculated ECD spectrum for withanolide S. We hence conclude that withanolide S is the major metabolite of 4BHWE in both insect species.

Since the product obtained is a pure diastereomer (only 5*α*‐hydroxylation was observed), and since the rearrangement leading to its formation as a multi‐step reaction is very unusual, we propose an enzymatically catalyzed mechanism, e.g., by an epoxide hydrolase. These enzymes have been suggested to be involved in insect detoxification of plant defensive compounds containing epoxides.^[^ ^31^ ^]^ An epoxide hydrolase has already been identified in *H. virescens*.^[^ ^32^ ^]^ The conversion does not change the polarity of the metabolite notably, as shown by the very slight difference in retention times in reversed‐phase chromatography, but results in changes in reactivity and stereochemistry.

In a previous study, the presence of an epoxide group on a withanolide was correlated with antifeedant activity on the generalist insect *Spodoptera littoralis*.^[^ ^33^ ^]^ It would be interesting to see whether 4BWHE deters feeding more than withanolide S. Any antifeedant activity of a compound likely acts on the gustatory receptors during consumption of the food; however, we observed withanolide S in the frass after the 4BHWE was consumed. Therefore, additional studies are needed before any conclusions about detoxification can be drawn.

The metabolic transformation described in this study cannot explain the aforementioned growth differences between *subflexa* and *virescens* when fed an artificial diet containing withanolides.^[^ ^11^ ^]^ Many other compounds may be involved in growth regulation, and in addition to the epoxide transformation of 4BHWE, there are many other metabolic steps. While we assume that the epoxide transformation is a necessary step in lowering the chemical reactivity of the diet, any physiological outcome must be interpreted in the context of the full spectrum of compounds in the diet. Future experimental work will help us to understand this complex system.

Whether 4BHWE or withanolide S has ecdysteroidal activity must also be the subject of further study. Ecdysteroids share the structural feature of a 5*β*‐methine together with a keto function in position 6 and hydroxylation in positions 3 and 22.^[^ ^34^, ^35^ ^–^ ^36^ ^]^ Neither withanolide S nor 4BHWE seem to have structural similarity to 20E (Figure S41, Supporting Information). An expanded study with a greater variety of withanolides and insect species will further our knowledge in this area.

## 4 Conclusion

In the present study, we investigated the metabolic transformation of 4BHWE by larvae of an insect species that routinely consumes withanolides and larvae of a closely related species that does not. A uniformly ^13^C‐labeled precursor was used as a substrate, which allowed unambiguous determination of the main metabolite in the insect frass. The ^13^C‐labeled compound was administered with an artificial diet, and the major transformation product, withanolide S, was recovered in both insect species. The precursor 4BHWE contains a reactive structural element, a β‐epoxide moiety, which was converted to a *trans*‐diol either by stepwise rearrangement, ring opening and hydride transfer, or, more likely, was transformed by an epoxide hydrolase. The substrate 4BHWE undergoes a significant structural change by conversion to withanolide S, which is an all‐*trans*‐fused triterpene derivative. The observed transformation can be interpreted as the opening of a reactive withanolide epoxide.

## Conflict of Interest

The authors declare no conflict of interest.

## Author Contributions

All authors accepted the responsibility for the content of the manuscript, consented to its submission, reviewed all the results, and approved the final version of the manuscript. CP isolated and purified 4HBWE and its transformation product withanolide S, recorded and analyzed LC‐MS, NMR, and ECD analytical data, calculated chemical structures and ECD spectra by molecular modelling. MPS initiated the study, planned, and conducted the feeding assays. FF supervised the isotopic labeling procedure. YP provided supervision and support. CP and MPS drafted the manuscript, and DGH revised the manuscript.

## Supporting information

Supplementary Material

## Data Availability

The data that support the findings of this study are available from the corresponding author upon reasonable request.
