# Supplementary material for: Metabolism of Epoxidised Withanolides by a Generalist and a Specialist Insect Species
Source: ChemistryOpen. 2025 Sep 11;14(12):e202500359. doi: 10.1002/open.202500359 (PMC12680557; doi:10.1002/open.202500359)
Supplement: Supplementary file 1 — Supplementary Material [file OPEN-14-e202500359-s001.pdf]

-

Supporting information

Content:

SI Figure 1: MCI gel CP20P fractionation scheme of the ^{13}C-labelled *P. peruviana* leaf extract

SI Figure 2: Second MPLC (C-18) fractionation scheme to purify ^{13}C-labelled 4β-hydroxywithanolide E (4BHWE)

SI Figure 3: HRMS-analytical data of the ^{13}C-labelled probe 4BHWE

SI Figure 4: Main fragment ions of 4BHWE with possible structures, pos. ionisation mode, ESI.

SI Figure 5: Fragment ion *m/z* 123.0804 with possible structure, pos. ionisation mode, ESI

SI Figure 6: Fragment ion *m/z* 169.0859 with possible structure, pos. ionisation mode, ESI

SI Figure 7: Fragment ions *m/z* 281.1536, *m/z* 299.1642, and *m/z* 317.1747 with possible structures, pos. ionisation mode, ESI

SI Figure 8: Fragment ions *m/z* 449.2323, *m/z* 467.2428, *m/z* 485.2534 with possible structures, and the Na^+ adduct *m/z* 525.2459, pos. ionisation mode, ESI

SI Figure 9: Structure of 4BHWE with chemical shifts

SI Figure 10: ^{1}H-NMR spectrum of 4BHWE

SI Figure 11: ^{13}C-NMR spectrum of 4BHWE

SI Figure 12: ^{1}H-^{1}H DQFCOSY of 4BHWE

SI Figure 13: ^{1}H-^{1}H ROESY of 4BHWE

SI Figure 14: ^{1}H-^{13}C HSQC of 4BHWE, full range

SI Figure 15: ^{1}H-^{13}C HSQC of 4BHWE, aliphatic range

SI Figure 16: ^{1}H-^{13}C HMBC of 4BHWE, full range

SI Figure 17: ^{1}H-^{13}C HSQC-TOCSY of 4BHWE, full range

SI Figure 18: HPLC-UV-HRMS chromatograms of feces samples

SI Figure 19: HPLC-UV-HRMS chromatogram of the combined feces extracts

SI Figure 20: HPLC-UV-HRMS chromatogram of the combined feces samples, detail

SI Figure 21: ^{1}H-NMR spectrum of withanolide S

SI Figure 22: ^{13}C-DEPTQ spectrum of withanolide S

SI Figure 23: ^{1}H-^{13}C HSQC spectrum of withanolide S, full range

SI Figure 24: ^{1}H-^{13}C HSQC spectrum of withanolide S, aliphatic range

SI Figure 25: 1,1-ADEQUATE spectrum of withanolide S, full range

SI Figure 26: 1,1-ADEQUATE spectrum of withanolide S, detail (7.0-3.5 (F2 ppm))

SI Figure 27: 1,1-ADEQUATE spectrum of withanolide S, detail (3.5-2.0 (F2 ppm))

SI Figure 28: 1,1-ADEQUATE spectrum of withanolide S, detail (2.4-1.8 (F2 ppm))

SI Figure 29: 1,1-ADEQUATE spectrum of withanolide S, detail (1.8-1.1 (F2 ppm))

SI Figure 30: ^{1}H-^{13}C HMBC spectrum of withanolide S, detail (2.0-1.1 (F2 ppm))

SI Figure 31: ^{1}H-^{13}C HSQC-TOCSY spectrum of withanolide S, detail (3.7-1.1 (F2 ppm))

SI Figure 32: ^{1}H-^{1}H ROESY spectrum of withanolide S, detail (1.4-1.1 (F2 ppm))

SI Figure 33: Graphical interpretation of the ROESY data from SI Figure 32

SI Figure 34: ^{1}H-^{1}H ROESY spectrum of withanolide S, detail (4.7-1.0 (F2 ppm))

SI Figure 35: Graphical interpretation of the ROESY data from SI Figure 34

SI Figure 36: Calculated ECD spectra of withanolide S and 4-deoxywithaperuvin

SI Figure 37: Experimental and calculated ECD spectra of withanolide S

SI Figure 38: Planar structure and the molecular model of withanolide S with stereo descriptors

SI Figure 39: Structure of withanolide S with chemical shifts

SI Figure 40: Possible metabolite structures ($C_{28}H_{40}O_8$) based on connectivity and oxygenation

SI Figure 41: Molecular models of 20E, 4BHWE, and withanolide S

SI Table 1: Chemical shift data of withanolide S and physaperuvin G

Biotage

Isolera™ Fractions Report PS_Physalis-13C

1.

**User**	Christian Paetz	**Cartridge**	SNAP Ultra C18 400g
**Sample Name**	PS_Physalis-13C	**Rack Type**	25x150 mm (45 ml)
**Date**	2021-Apr-16 11.14	**Max Fraction Volume**	45 ml
**Method**		**Solvent A**	Water 0.2% FA
**Detection Mode**	Lambda-all	**Solvent B**	Acetonitrile
**UV1 (Monitor)**	230 nm (Red)		
**UV2 (Monitor)**	254 nm (Black)		
**Lambda-all (Collect)**	(Brown)		

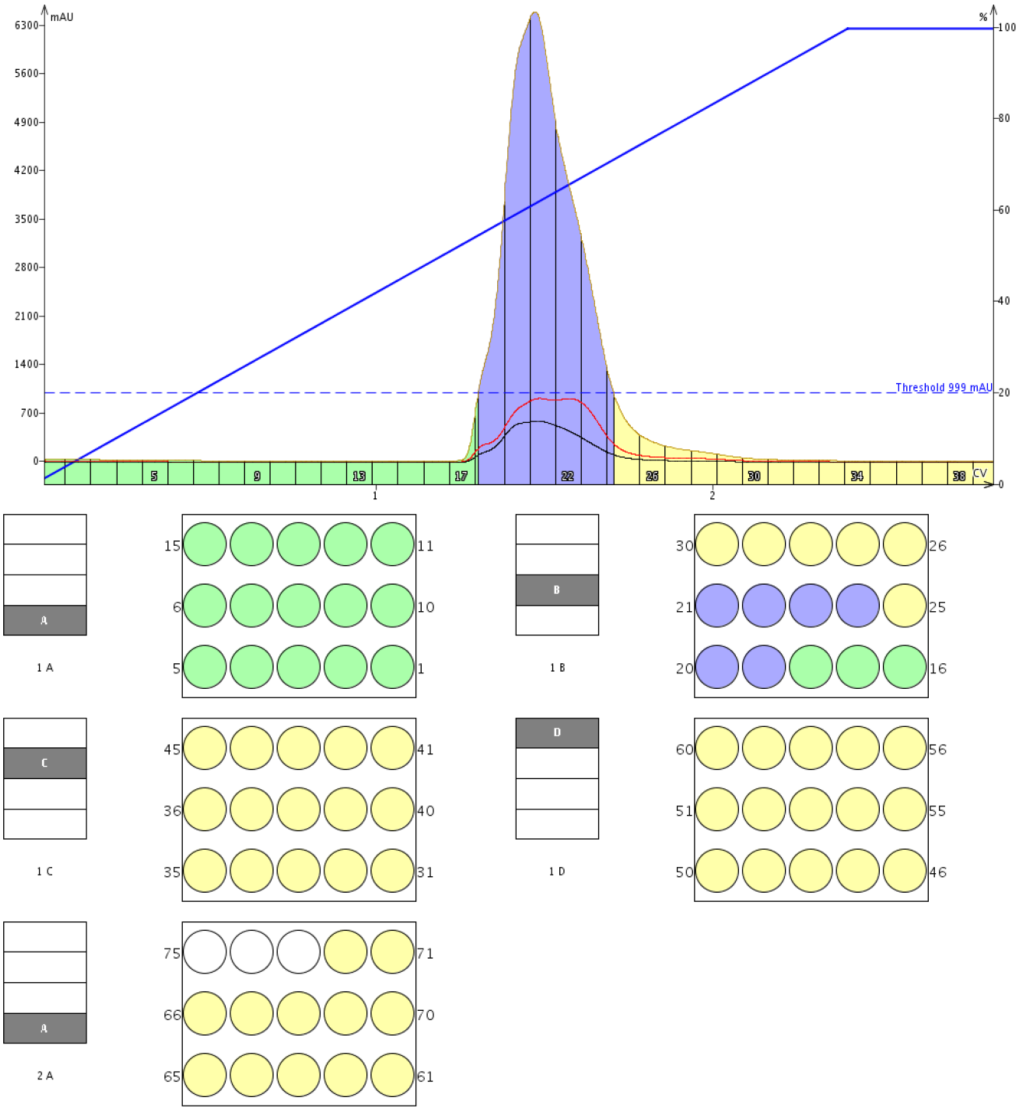

SI Figure 2: MCI gel CP20P fractionation of the defatted ^{13}C-labelled *Physalis peruviana* leaf extract.

Biotage **Isolera™ Fractions Report 13C_Phys_MeOH_1.12-2.8** 1.

**User**	Christian Paetz	**Cartridge**	SNAP Ultra C18 12g
**Sample Name**	13C_Phys_MeOH_1.12-2.8	**Rack Type**	16x150 mm
**Date**	2021-Jun-25 13.24	**Max Fraction Volume**	20 ml
**Method**		**Solvent A**	Water 0.2% FA
**Detection Mode**	Lambda-all	**Solvent B**	Acetonitrile
**UV1 (Monitor)**	230 nm (Red)		
**UV2 (Monitor)**	254 nm (Black)		
**Lambda-all (Collect)**	(Brown)		

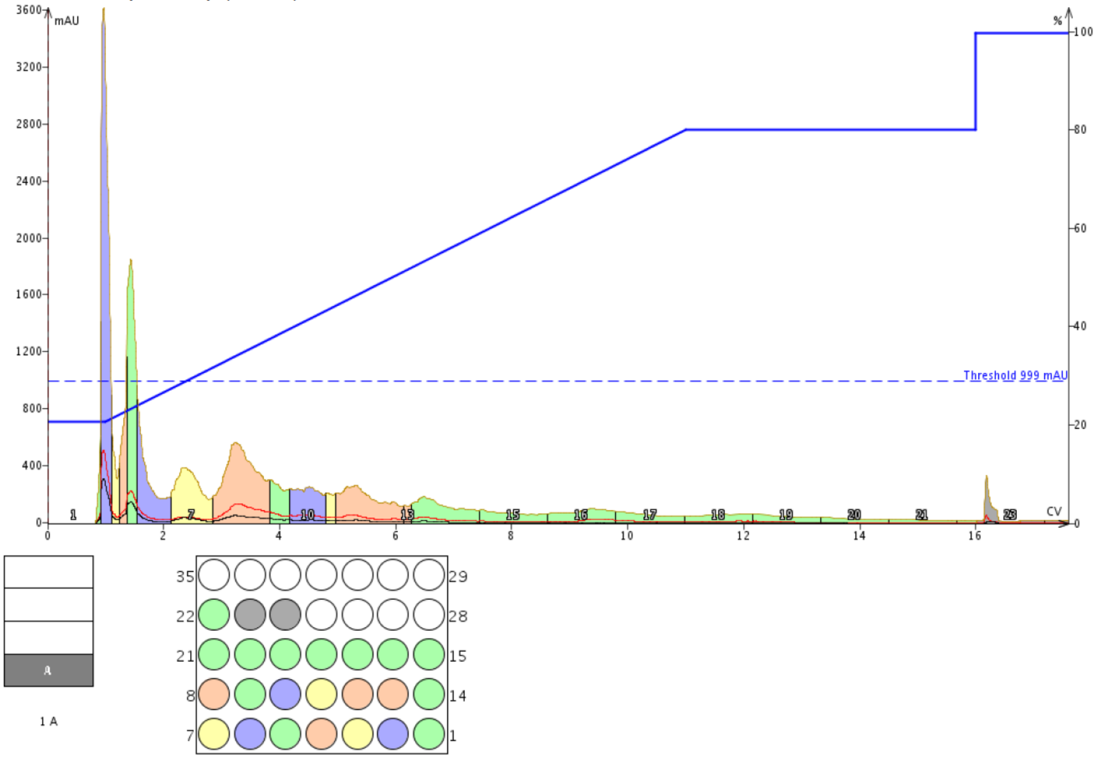

SI Figure 2: Second MPLC fractionation scheme (C-18) to purify ^{13}C-labelled 4β-hydroxywithanolide E (4BHWE).

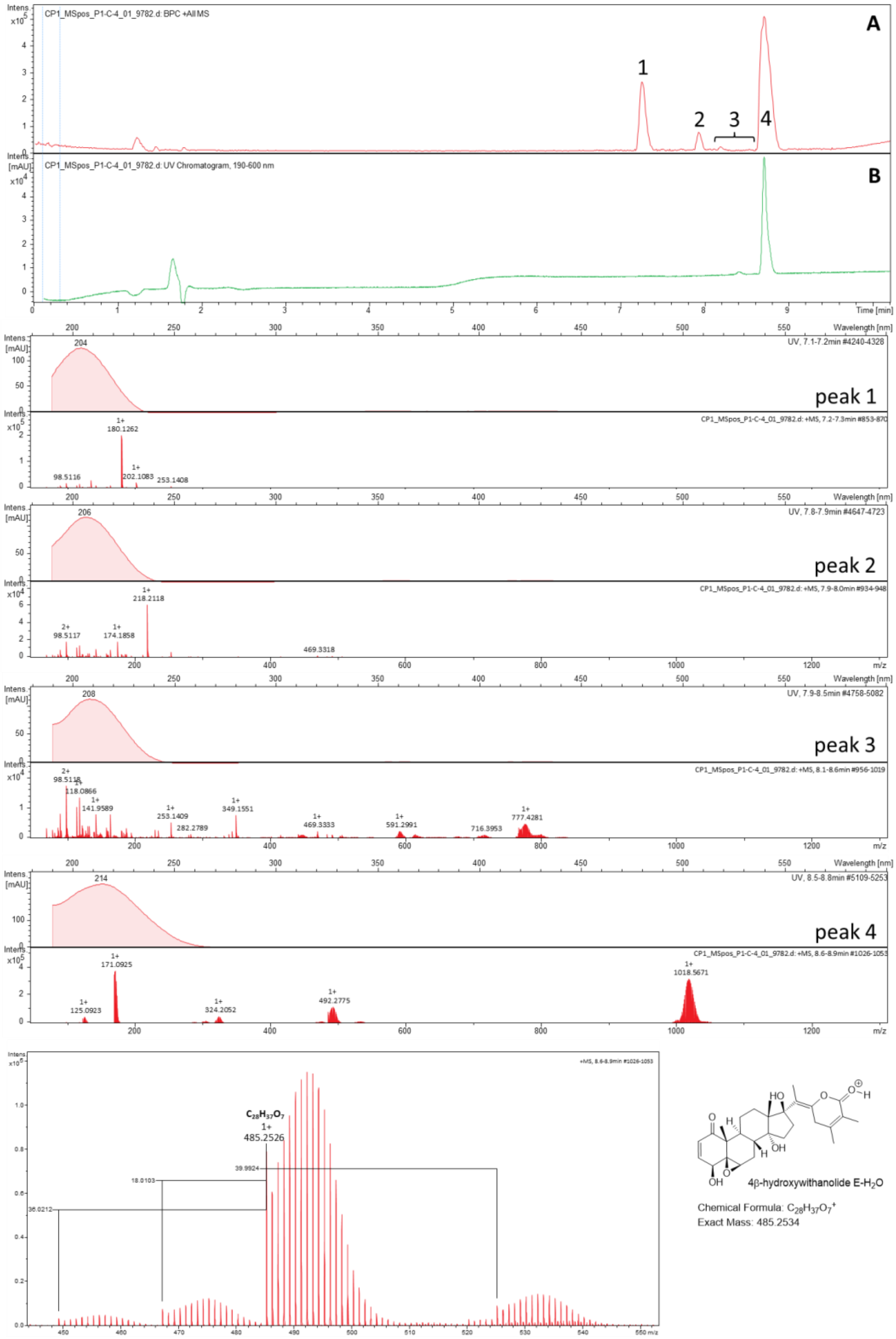

SI Figure 3: HRMS-analytical data of the ^{13}C-labelled probe 4BHWE. **A**) Positive mode HR-ESI-MS base peak chromatogram of the ^{13}C-labelled probe 4BHWE. **B**) UV trace (190-600 nm) of 4BHWE. The base peak chromatogram shows two additional peaks (peak 1 & peak 2) having no isotopologue pattern. The range 7.9 to 8.5 min contains ^{13}C labelled impurities (peak 3). Peak 4 represents the probe 4BHWE.

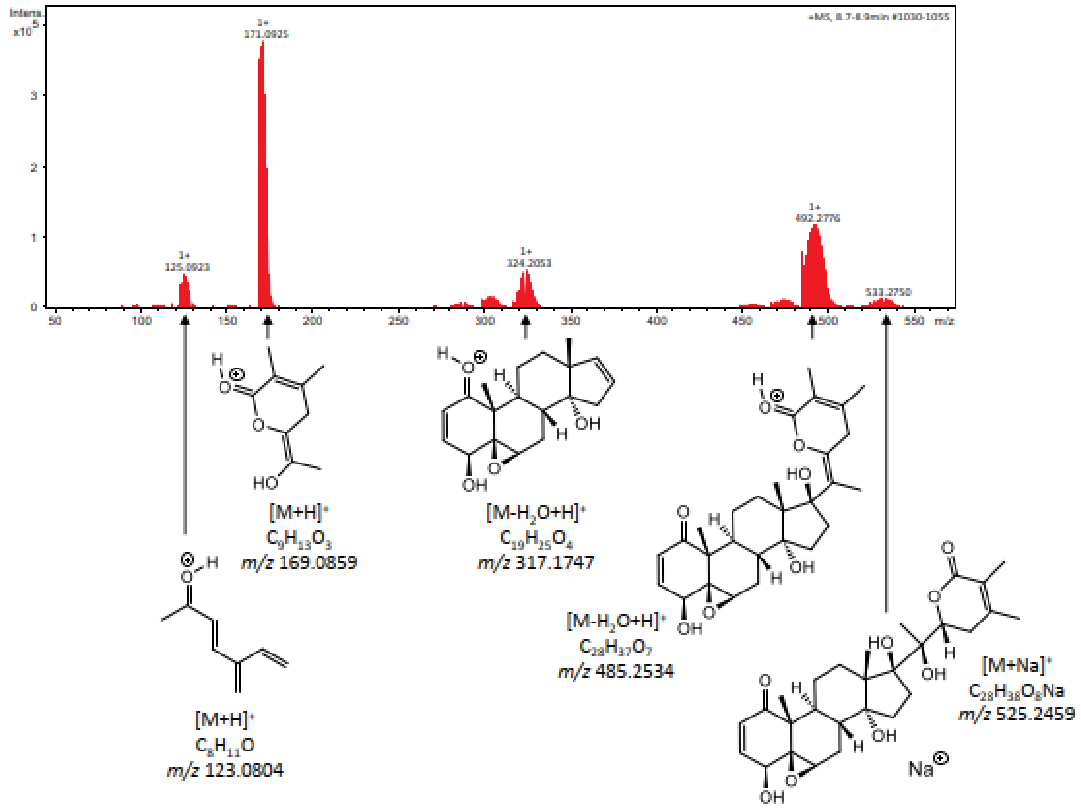

SI Figure 4: Main fragment ions of 4BHWE with possible structures, pos. ionisation mode, ESI.

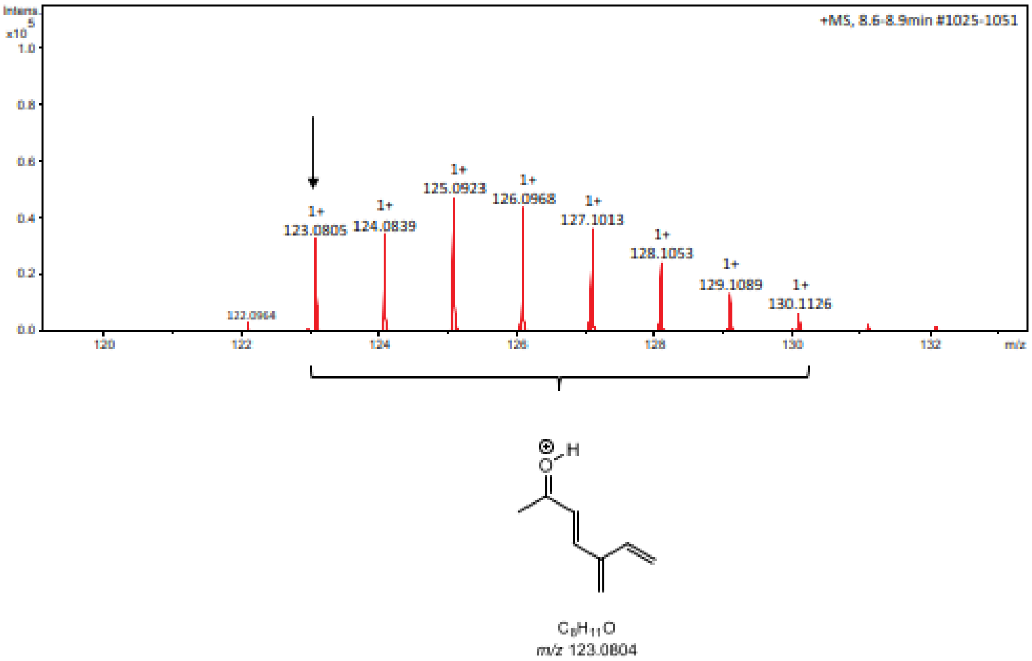

SI Figure 5: Fragment ion *m/z* 123.0804 with possible structure, pos. ionisation mode, ESI.

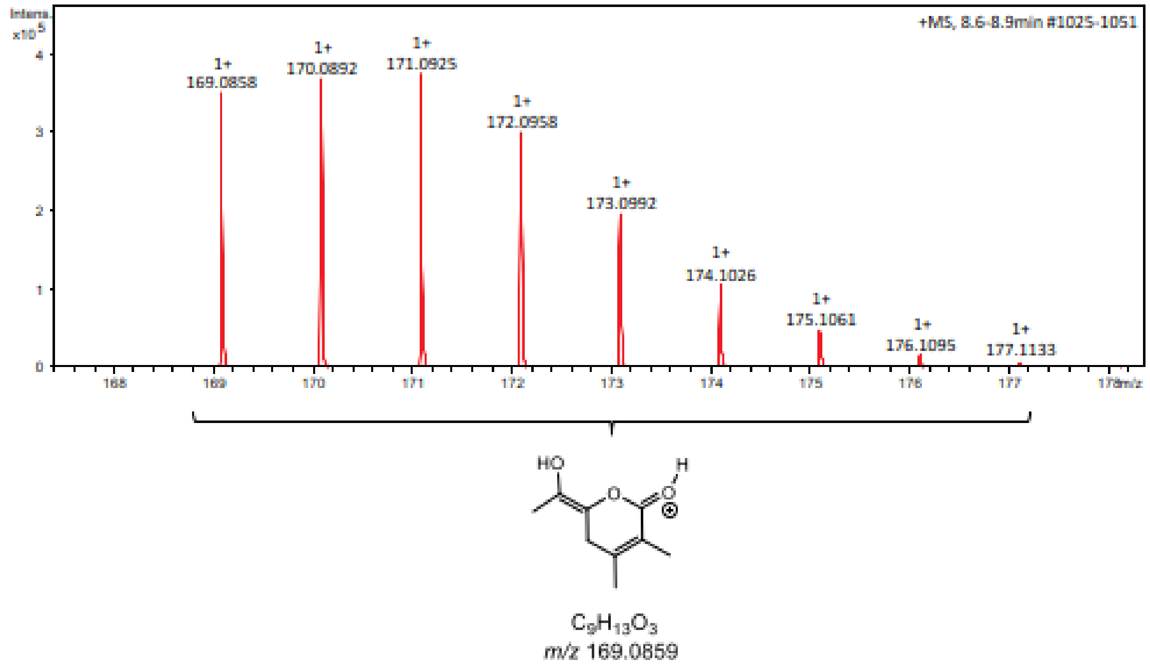

SI Figure 6: Fragment ion *m/z* 169.0859 with possible structure, pos. ionisation mode, ESI.

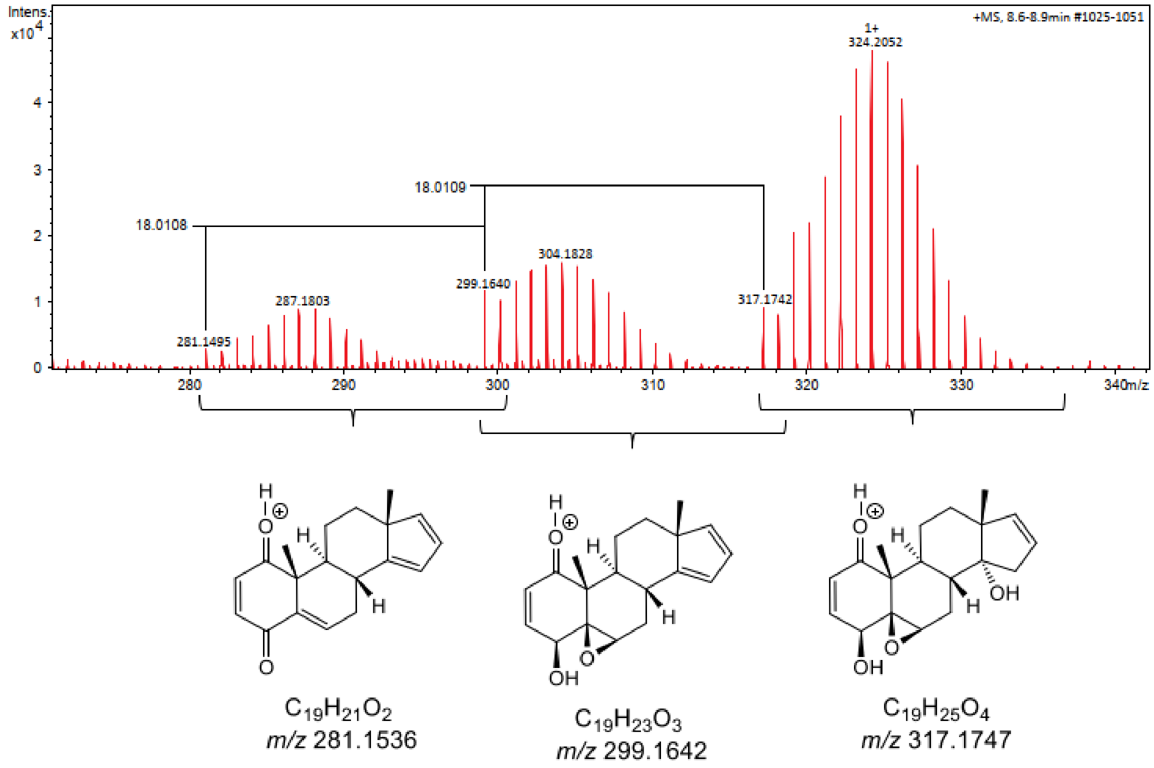

SI Figure 7: Fragment ions *m/z* 281.1536, *m/z* 299.1642, and *m/z* 317.1747 with possible structures, pos. ionisation mode, ESI.

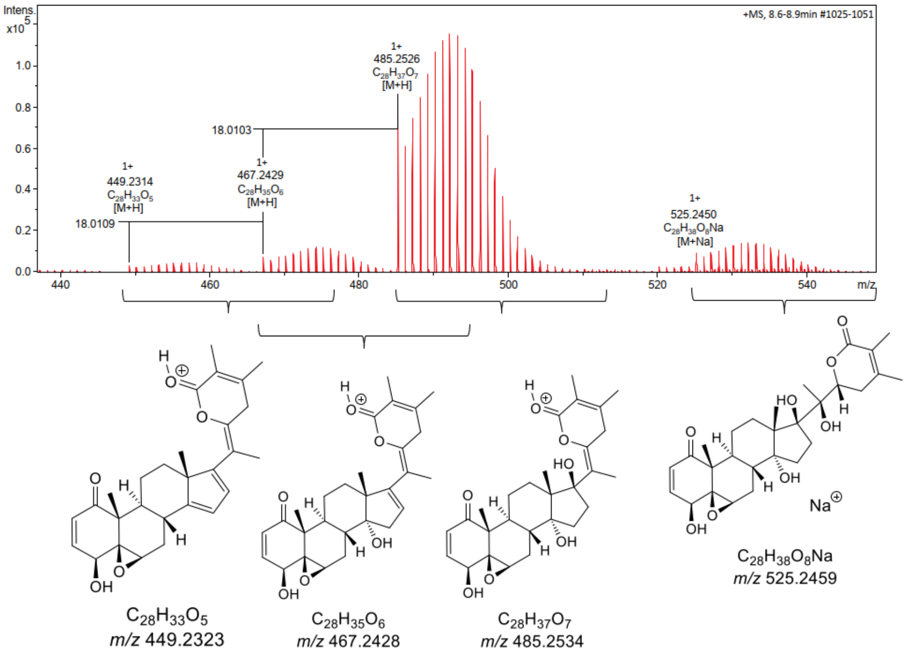

SI Figure 8: Fragment ions *m/z* 449.2323, *m/z* 467.2428, *m/z* 485.2534 with possible structures, and the Na^+ adduct *m/z* 525.2459, pos. ionisation mode, ESI.

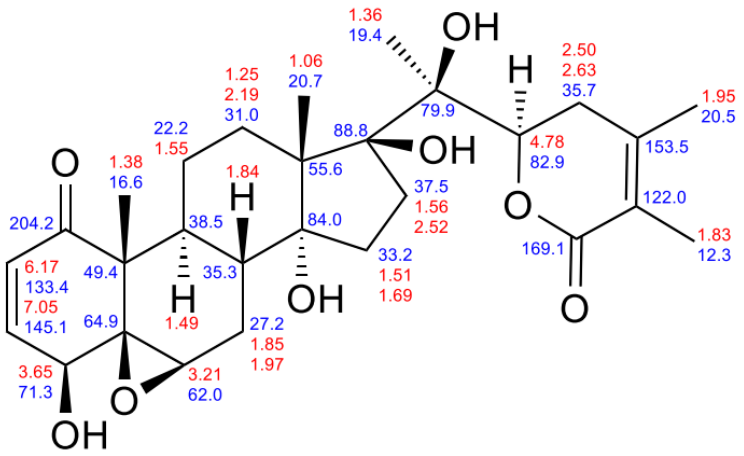

SI Figure 9: Structure of 4BHWE with chemical shifts (red δ_H, blue δ_C) in MeOH-d_3, 500 MHz. The data are in agreement with (24).

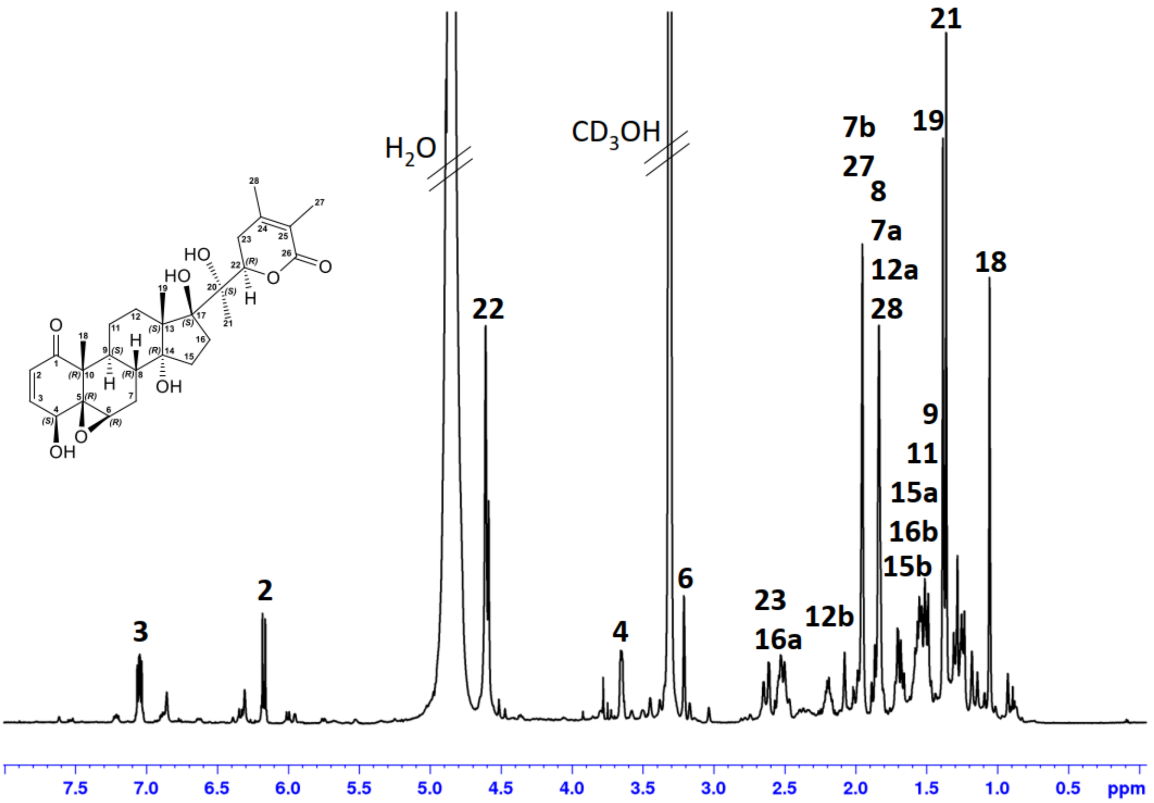

SI Figure 10: ^{1}H-NMR spectrum of the probe 4BHWE (500 MHz, 690 µg in 600 µl MeOH-d_3) produced by incubation of *P. peruviana* in [^{13}C]CO_2 atmosphere. The compound was isolated by chromatography from ^{13}C-labelled plant material. Bold numbers above the signals indicate the position in the molecule.

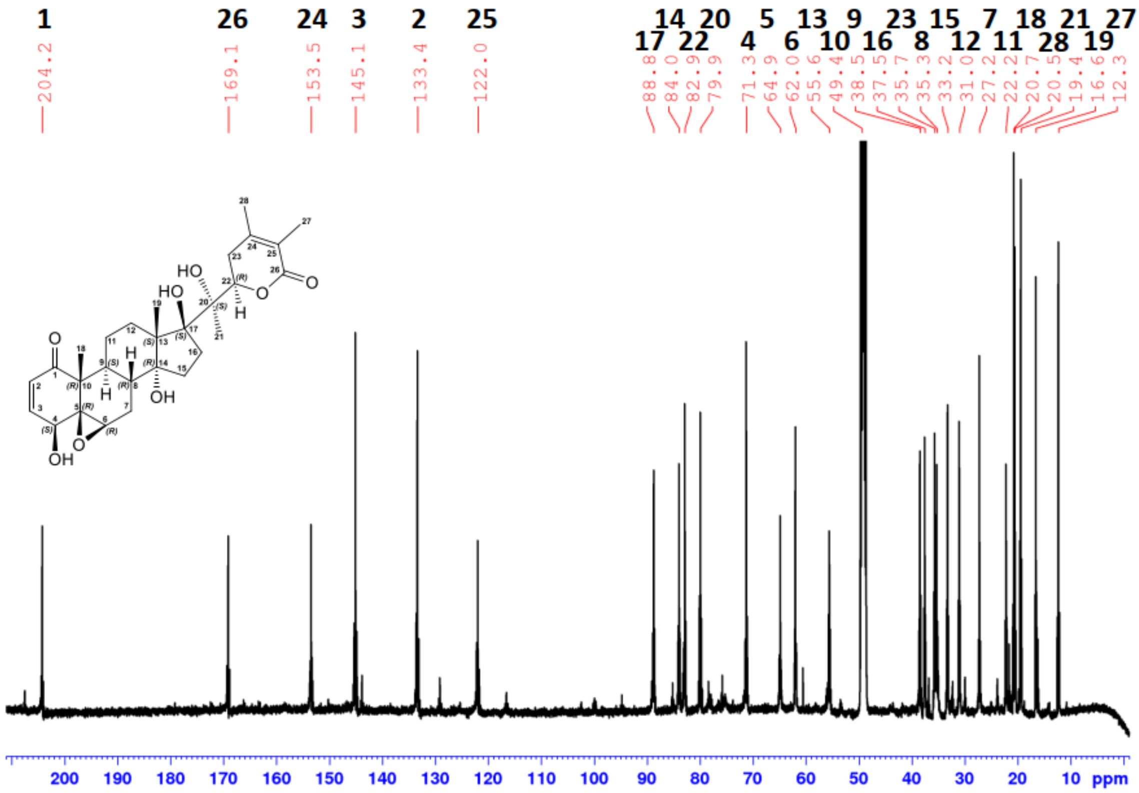

SI Figure 11: ^{13}C-NMR spectrum of the probe 4BHWE (125 MHz, 690 µg in 600 µl MeOH-d_3). The broadening at the base of each signal is due to ^{13}C satellite signals, indicating uniform ^{13}C labelling throughout the molecule. Bold numbers above the shifts indicate the position in the molecule.

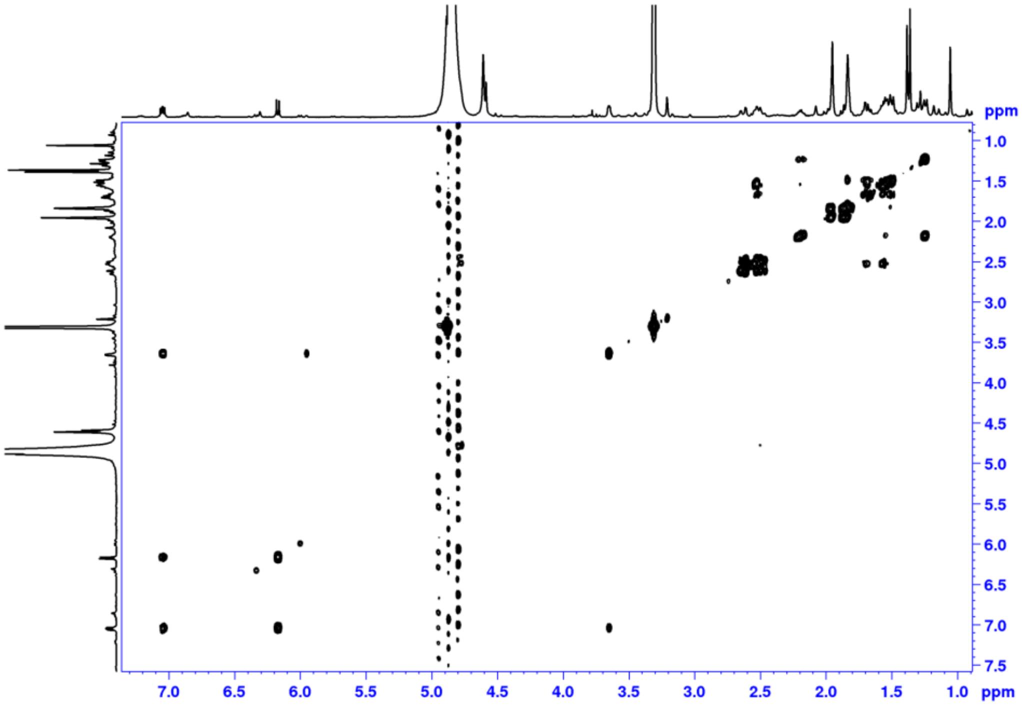

SI Figure 12: ^{1}H-^{1}H DQFCOSY of 4BHWE (690 µg in 600 µl MeOH-d_3).

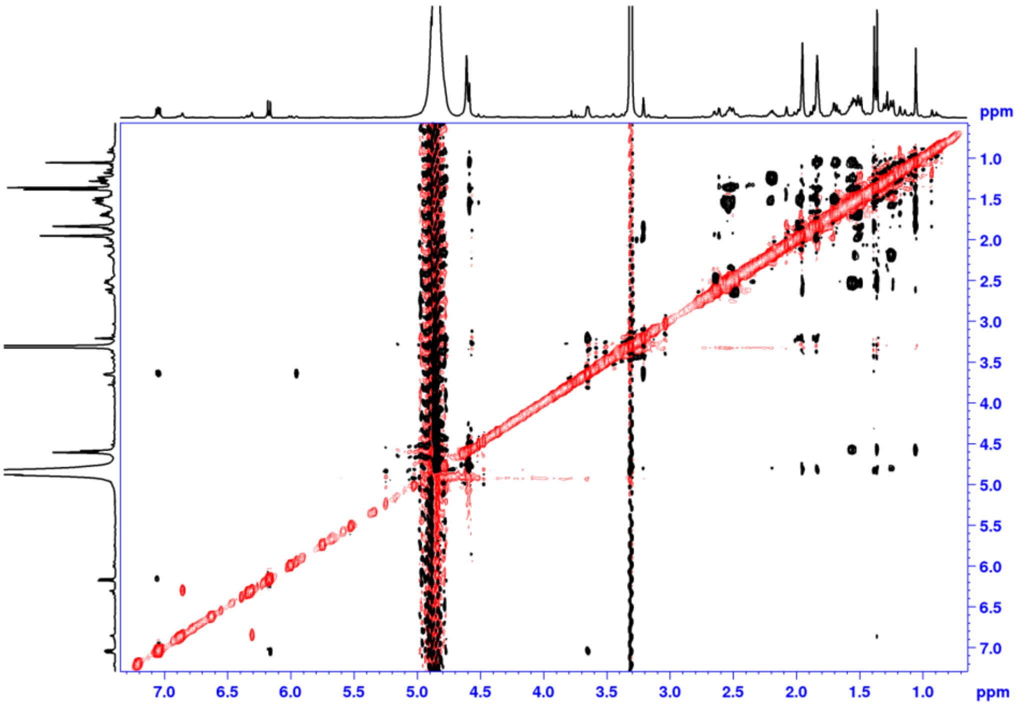

SI Figure 13: ^{1}H-^{1}H ROESY of 4BHWE (690 µg in 600 µl MeOH-d_3).

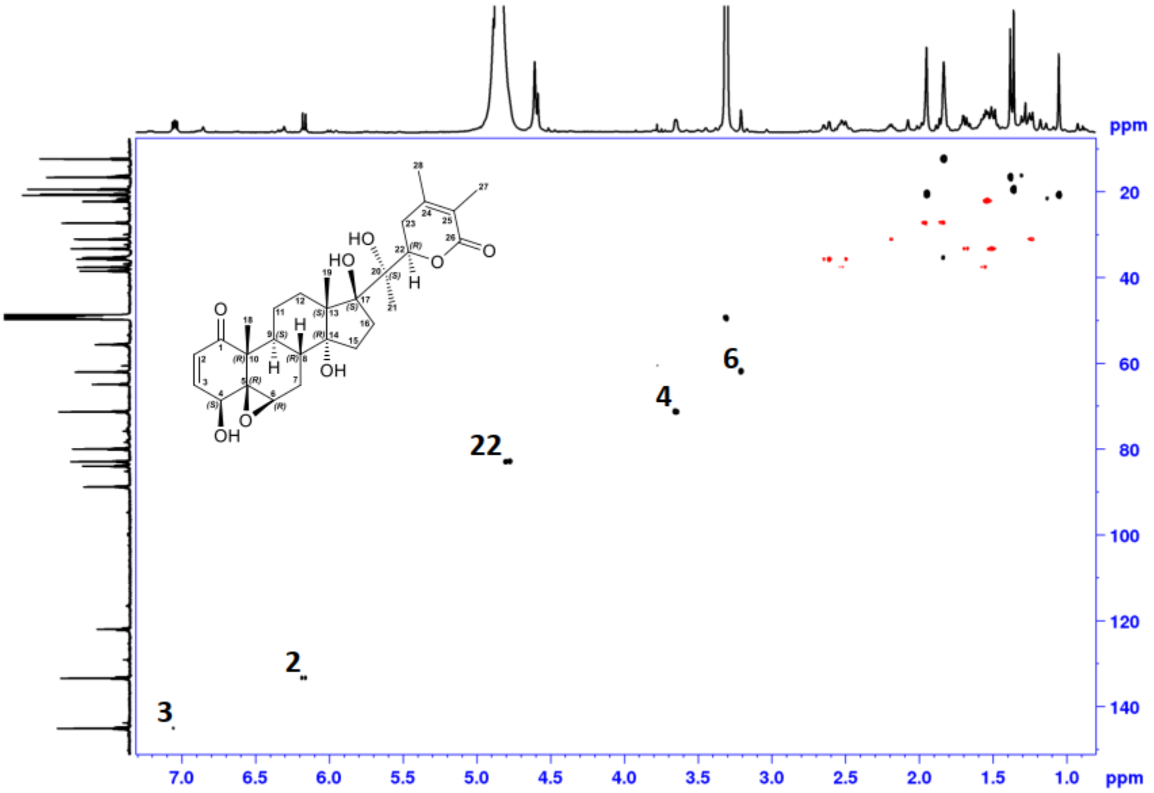

SI Figure 14: ^{1}H-^{13}C HSQC spectrum of 4BHWE (full range, 690 µg in 600 µl MeOH-d_3). Bold numbers above the signals (δ_H 7.5 to 3.0 in F2) indicate the position in the molecule.

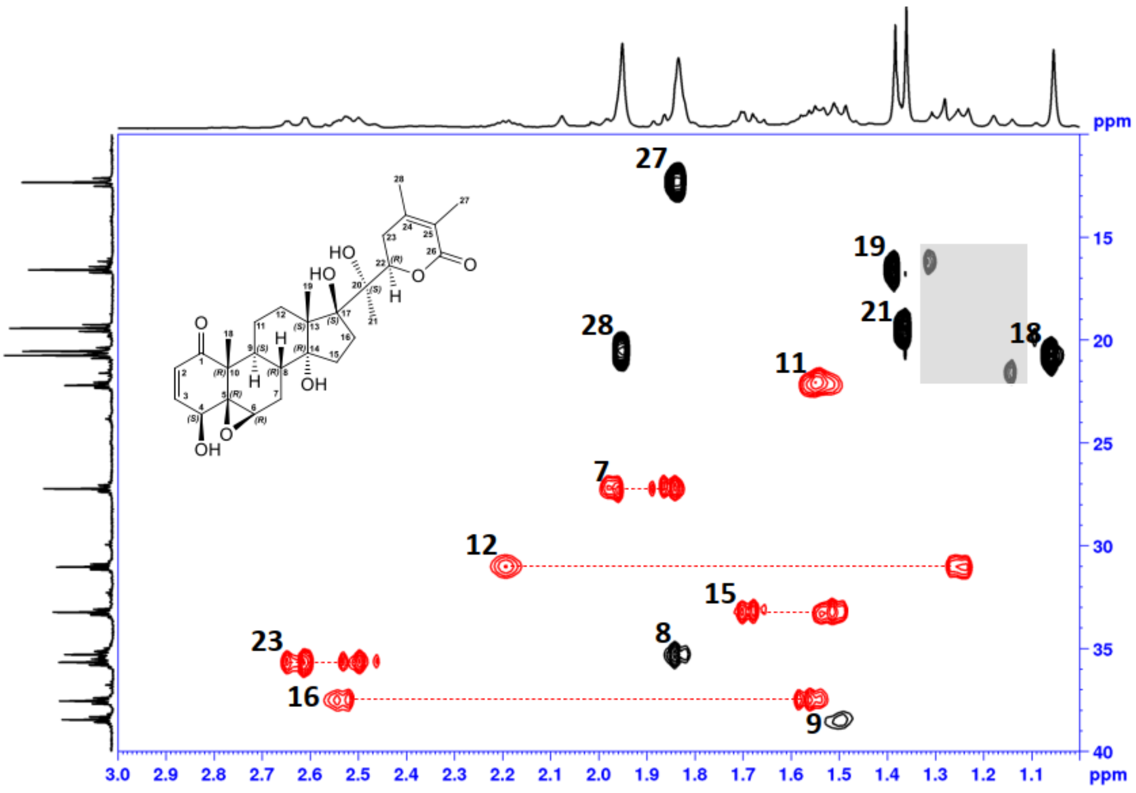

SI Figure 15: ^{1}H-^{13}C HSQC spectrum of 4BHWE (aliphatic range, 690 µg in 600 µl MeOH-d_3). Bold numbers above the signals indicate the position in the molecule. The gray rectangle covers signals of an impurity.

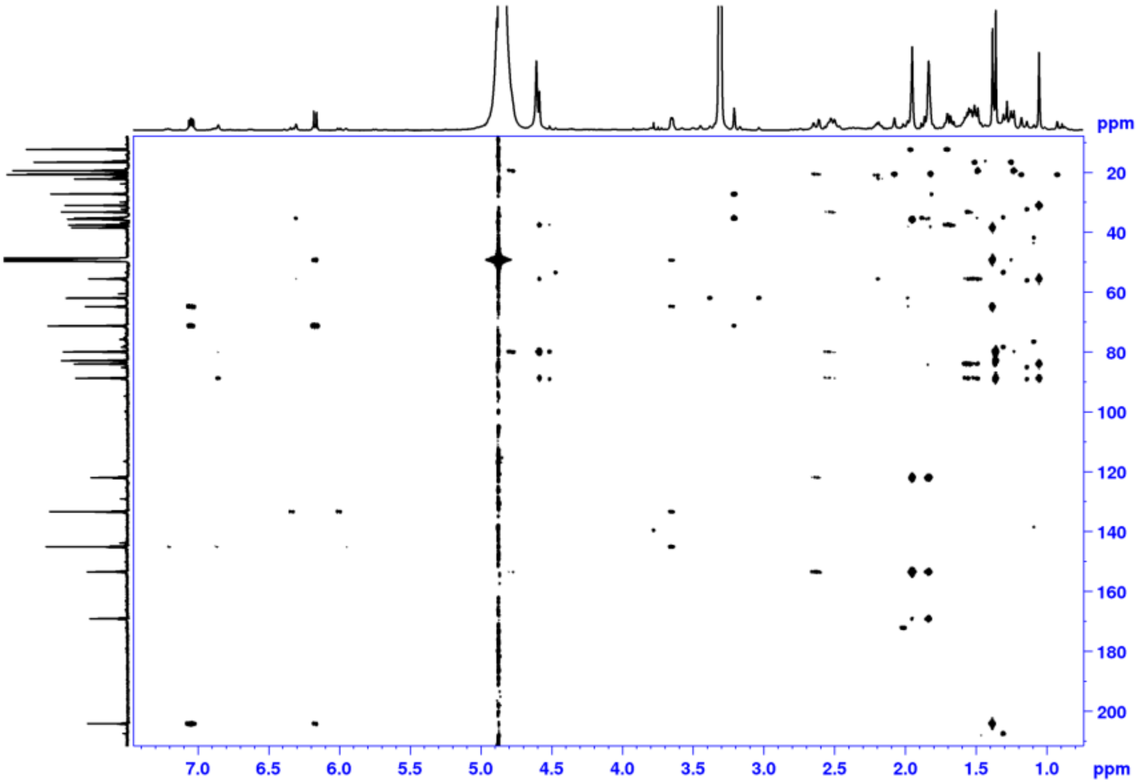

SI Figure 16: ^{1}H-^{13}C HMBC spectrum of 4BHWE (full range, 690 µg in 600 µl MeOH-d_3).

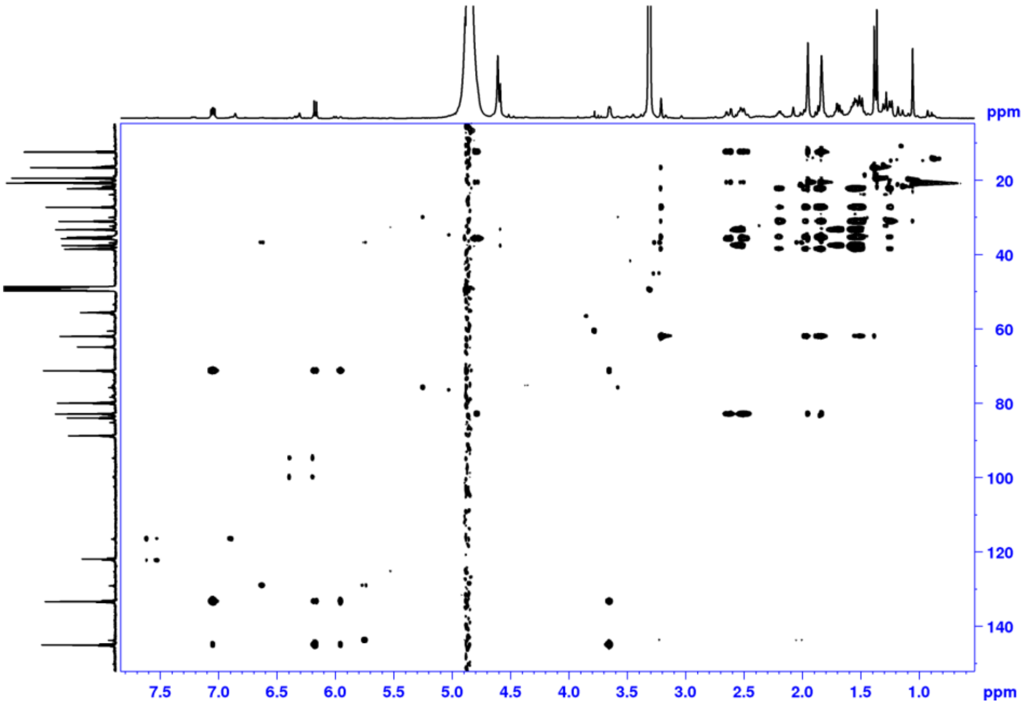

SI Figure 17: ^{1}H-^{13}C HSQC-TOCSY spectrum of 4BHWE (full range, 120 ms mixing time, 690 µg in 600 µl MeOH-d_3).

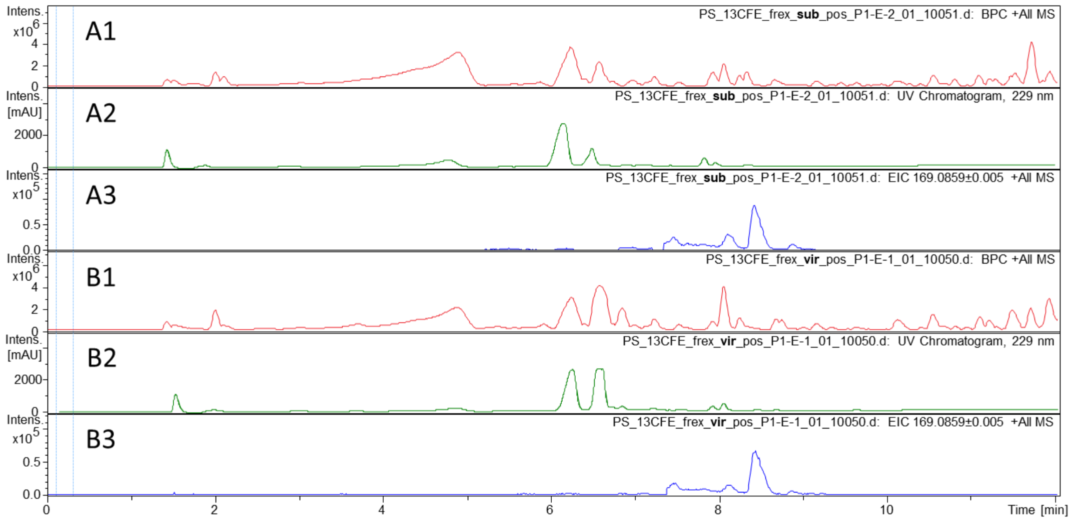

SI Figure 18: HPLC-UV-HRMS chromatograms of feces samples from *H. subflexa* (A1-A3) and *H. virescens (B1-B3)* after ingestion of 4BHWE. The figure shows the base peak (A1/B1 in red) and the UV chromatograms at 229 nm (A2/B2 in green). The extracted ion chromatograms (A3/B3 in blue) show the traces of withanolide compounds in both samples. Traces A3 and B3 are almost identical in composition and intensity. Therefore, the samples were pooled for the isolation and identification of the main compound.

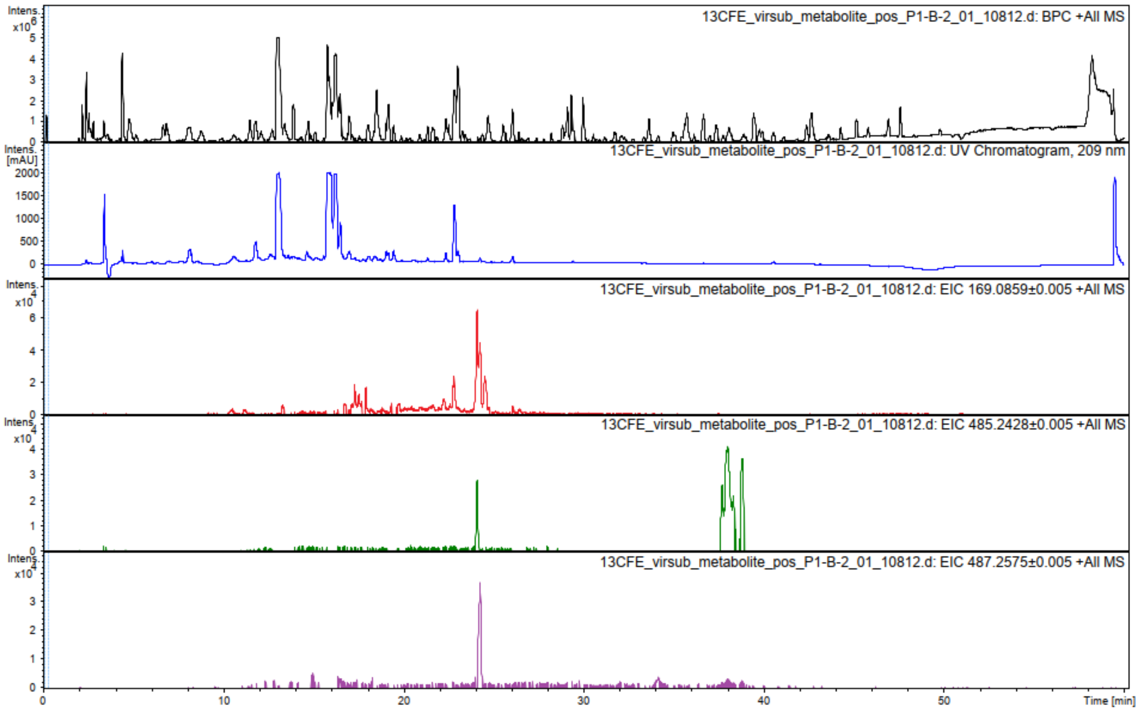

SI Figure 19: HPLC-UV-HRMS chromatogram of the combined feces extracts pooled from the two species after ingestion of 4BHWE. For separation, a Macherey-Nagel phenyl-hexyl column was used.

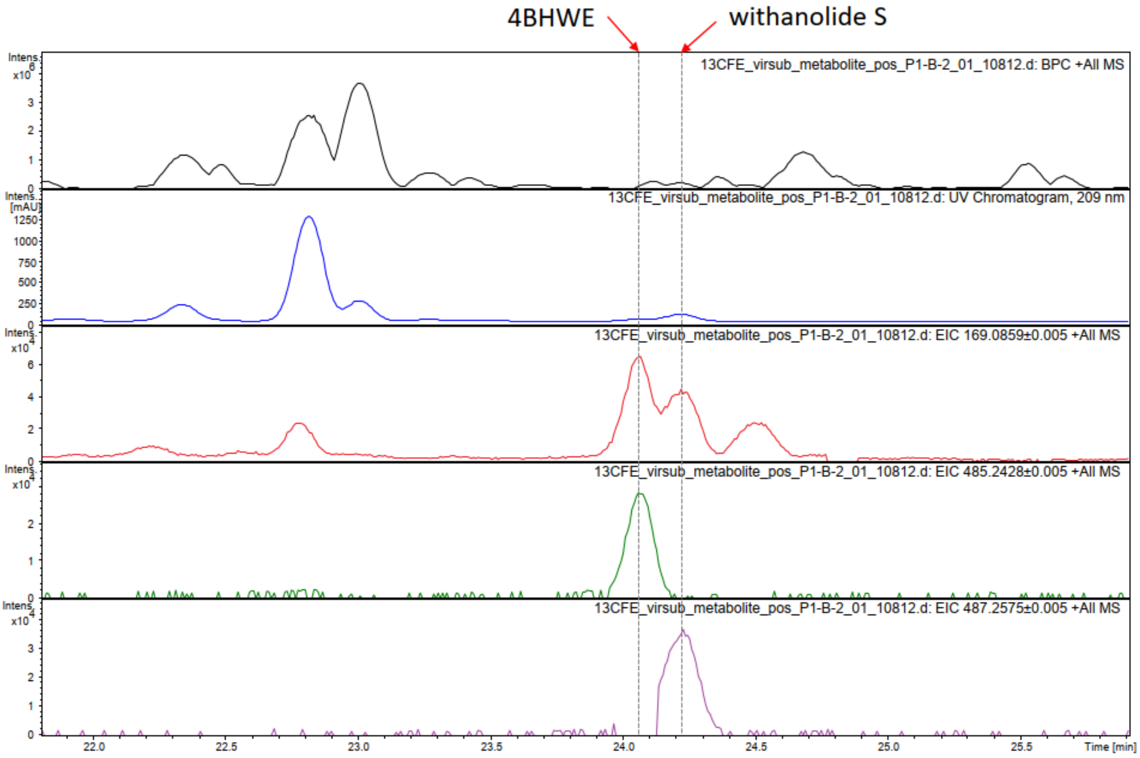

SI Figure 20: Detail of the HPLC-UV-HRMS chromatogram of the combined feces extracts after ingestion of 4BHWE. Separation on a MN phenyl-hexyl column.

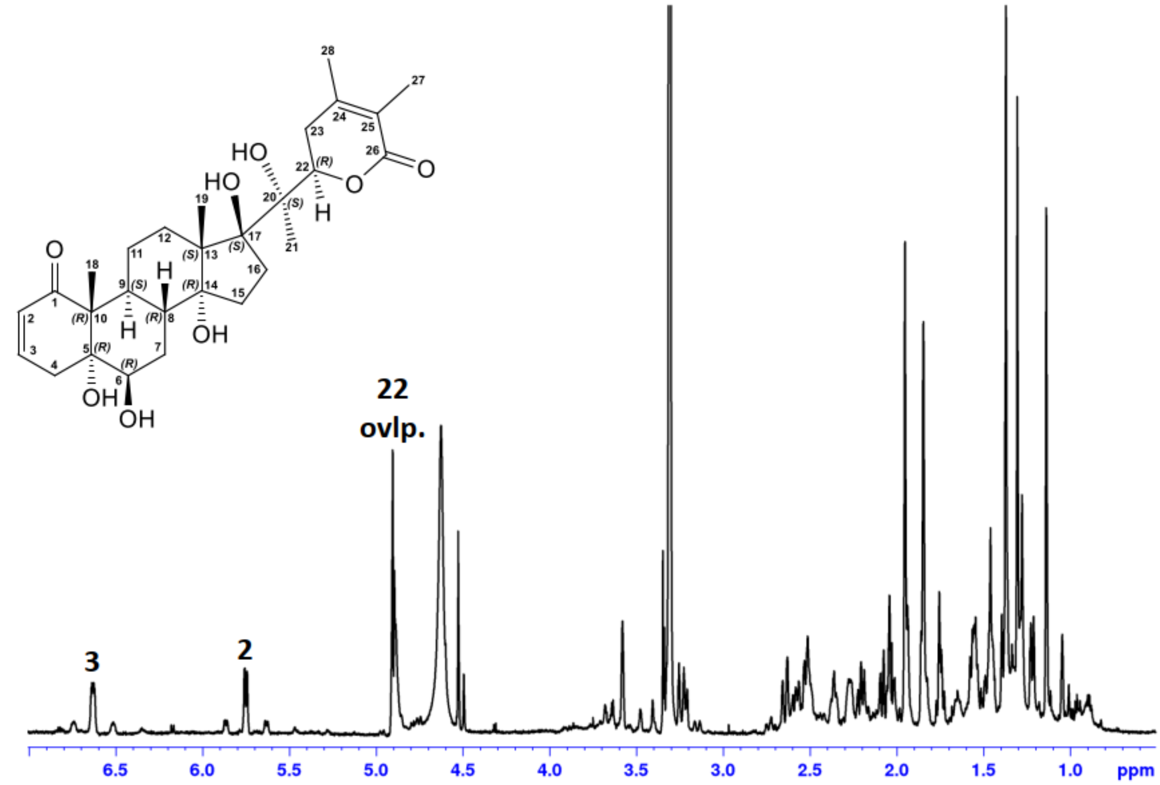

SI Figure 21: ^{1}H-NMR spectrum of the metabolite withanolide S (700 MHz, 130 µg in 30 µl MeOH-d_3) isolated from the combined feces extracts after feeding with ^{13}C-labelled 4BHWE. Bold numbers above the signals indicate the position in the molecule.

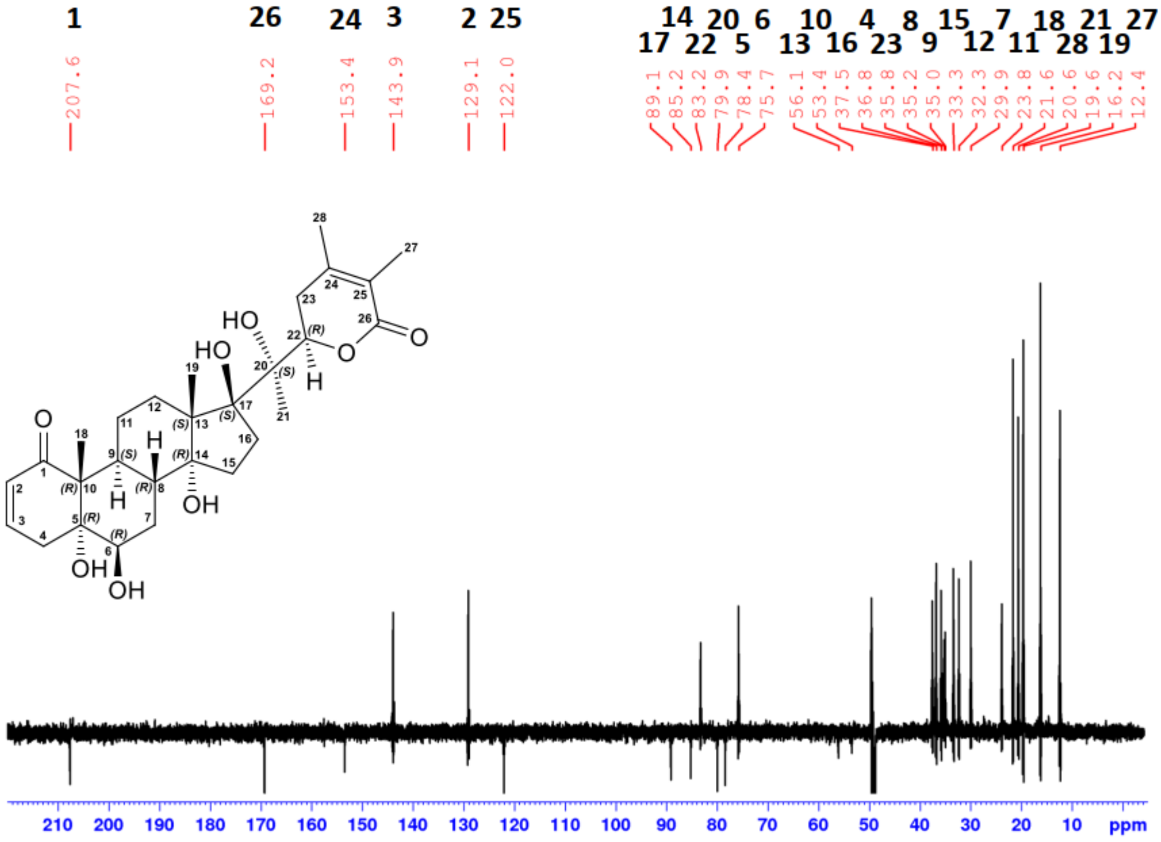

SI Figure 22: ^{13}C-DEPTQ-NMR spectrum of the metabolite withanolide S (175 MHz, 130 µg in 30 µl MeOH-d_3). Opposite phase signals in the aliphatic range result from distortions caused by ^{13}C labelling. Bold numbers above the shifts indicate the position in the molecule.

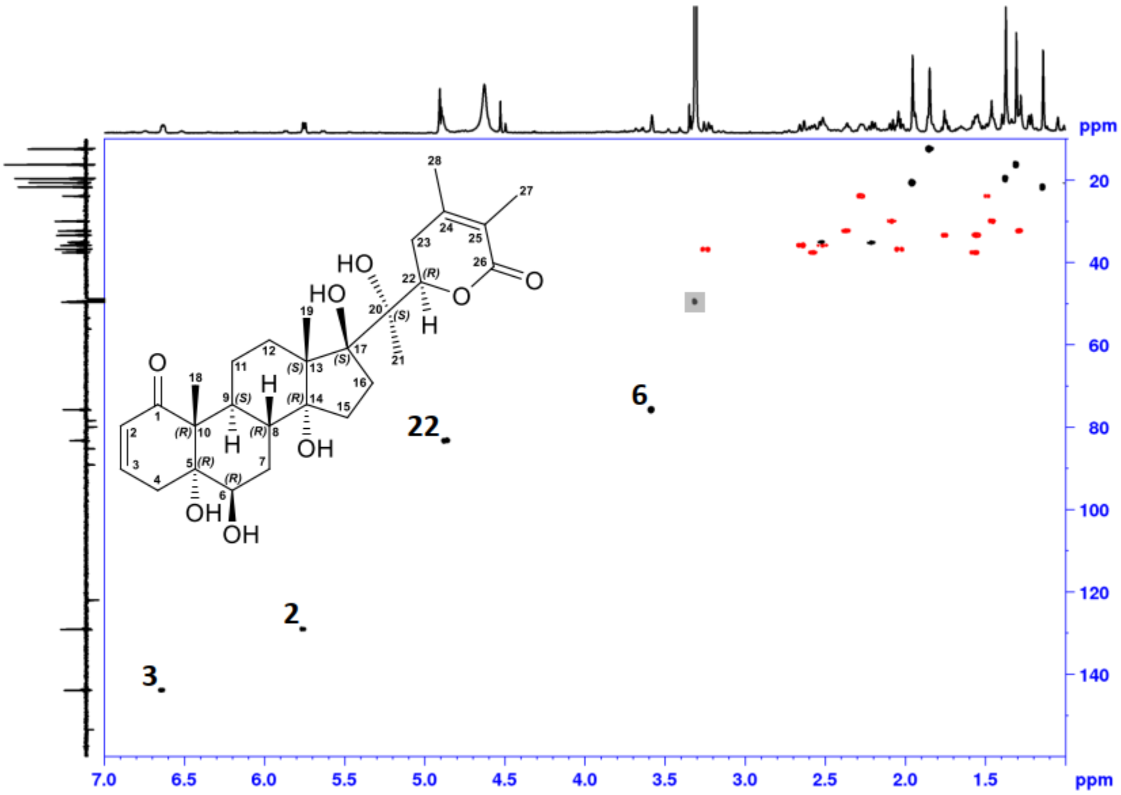

SI Figure 23: Full range phase edited ^{1}H-^{13}C HSQC spectrum of the metabolite withanolide S (130 µg in 30 µl MeOH-d_3). Numbers next to signals indicate the position in the molecule. The solvent signal is covered by a grey rectangle.

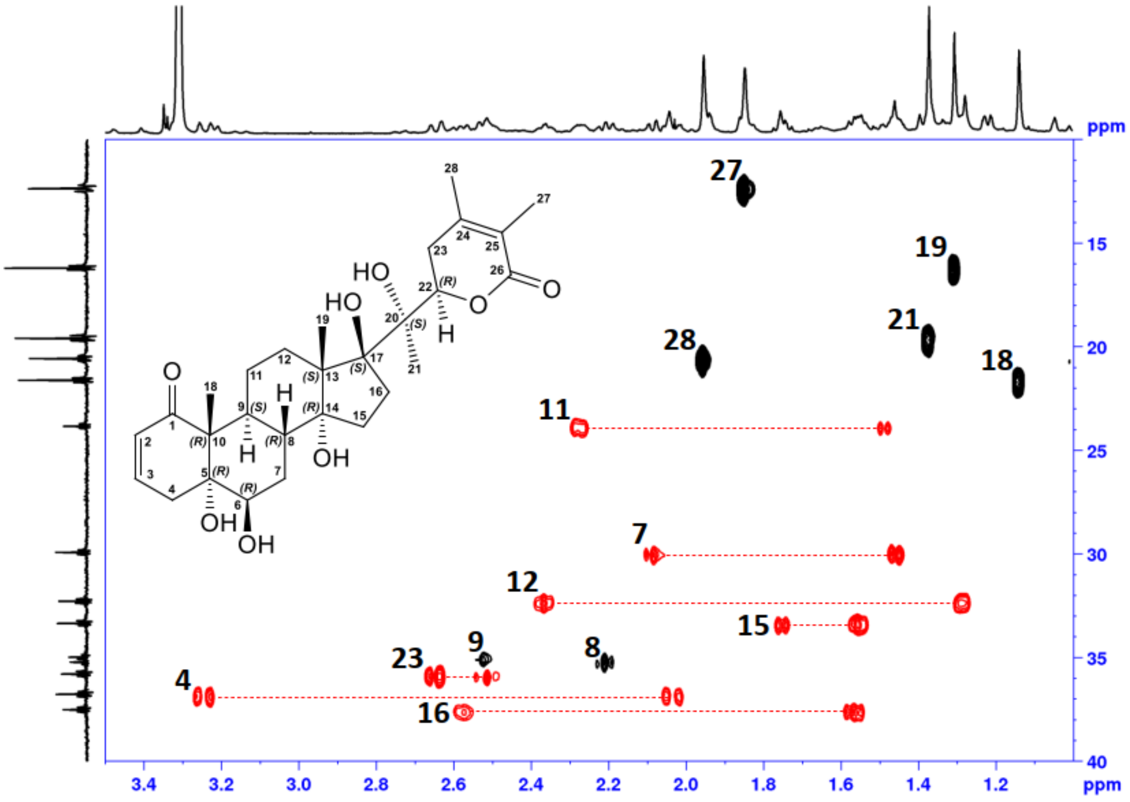

SI Figure 24: Detail of the edited ^{1}H-^{13}C HSQC spectrum of the metabolite withanolide S (130 µg in 30 µl MeOH-d_3). Numbers next to signals indicate the position in the molecule. Methylene signals are connected by a dotted line for better identification.

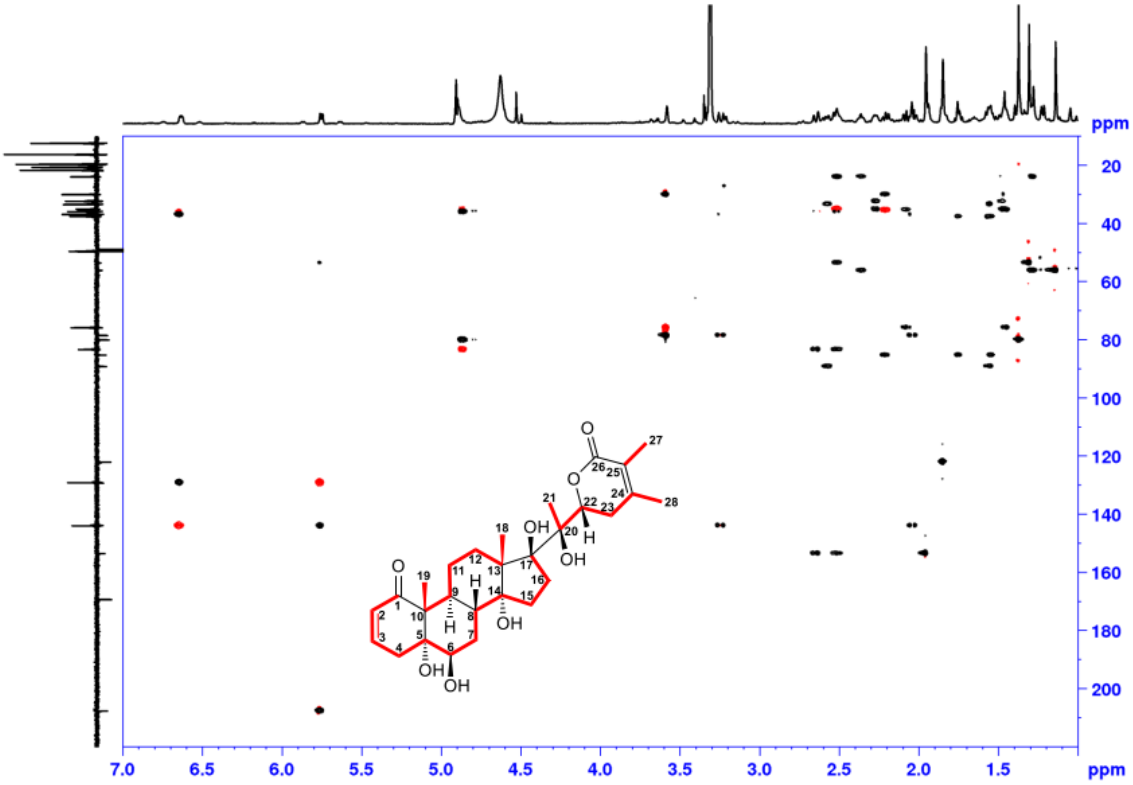

SI Figure 25: Full range phase edited and refocused 1,1-ADEQUATE spectrum of the metabolite withanolide S (130 µg in 30 µl MeOH-d_3). Red signals indicate $^1J_{CH}$ resonances. The formula shows the extractable connectivity information as red bonds.

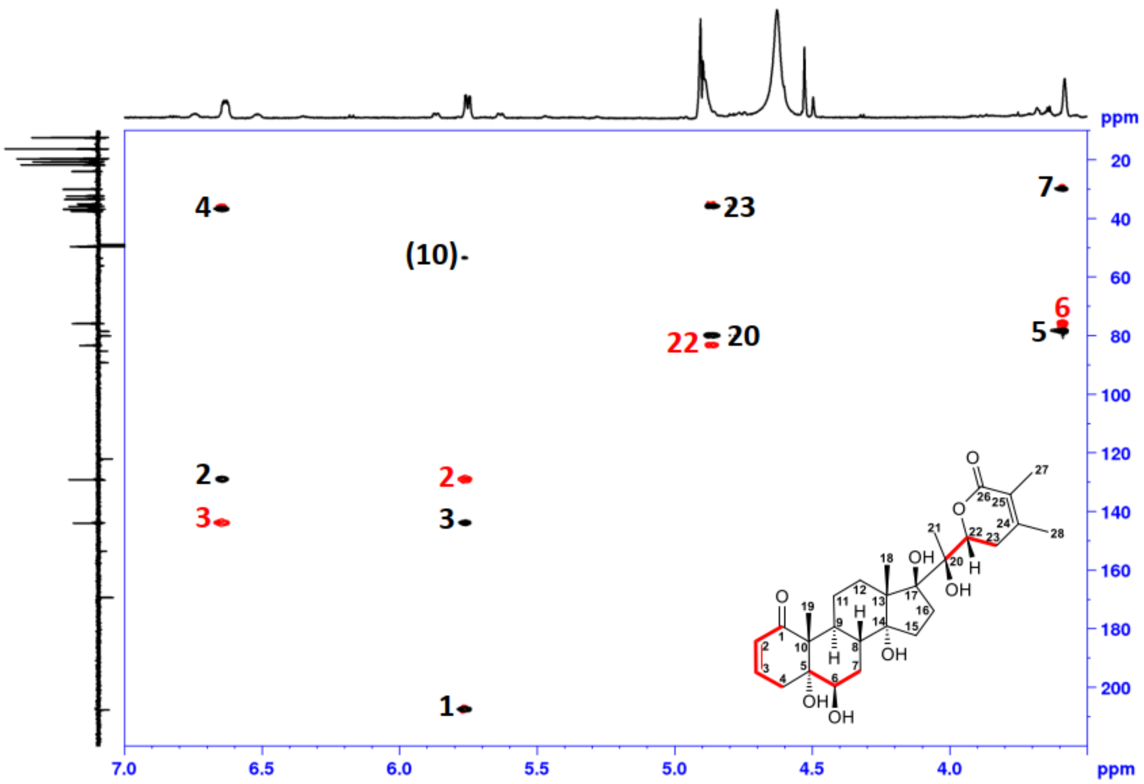

SI Figure 26: Detail of the phase edited and refocused 1,1-ADEQUATE spectrum of the metabolite withanolide S (130 µg in 30 µl MeOH-d_3). Red signals indicate $^1J_{CH}$ resonances, numbers next to signals indicate the position in the molecule. The formula shows the connectivity information as red bonds.

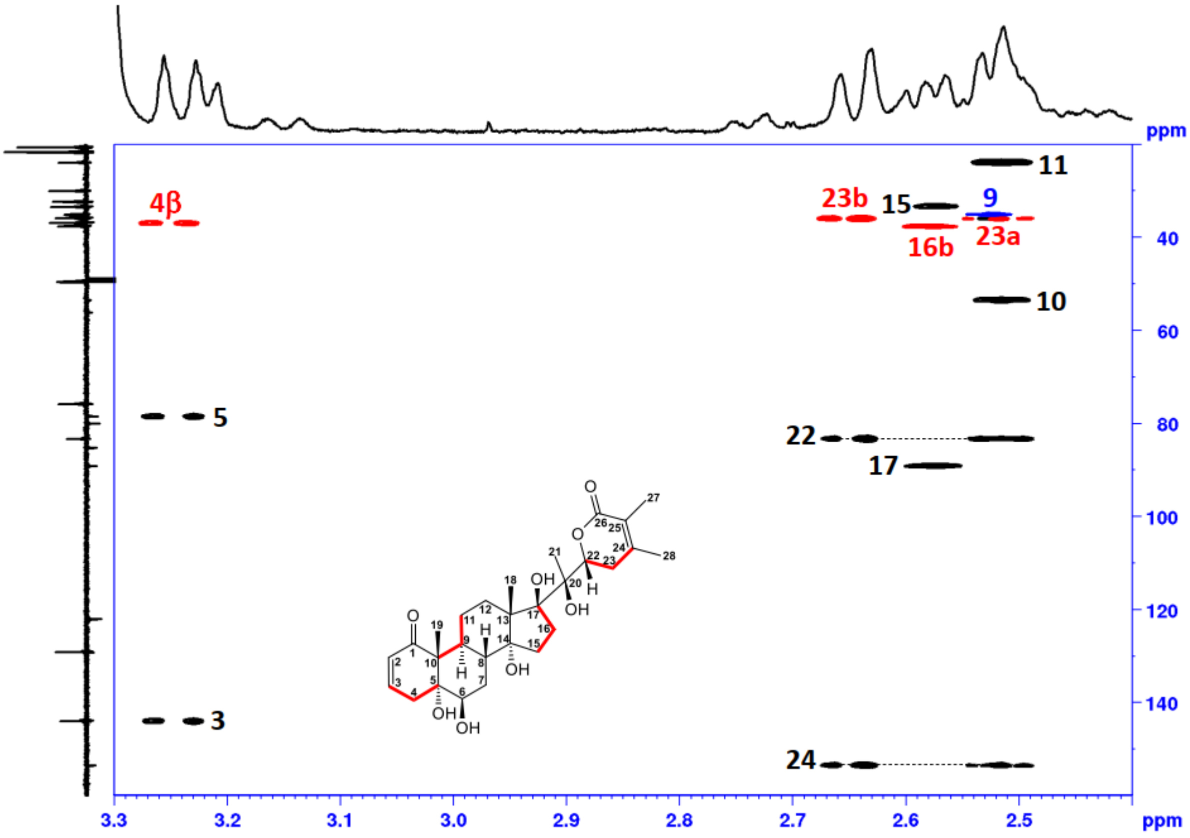

SI Figure 27: Detail of the phase edited and refocused 1,1-ADEQUATE spectrum of the metabolite withanolide S superimposed with the ^{1}H-^{13}C HSQC of the same spectral range (130 µg in 30 µl MeOH-*d*$_3$). Red and blue signals indicate $^{1}J_{CH}$ resonances, numbers next to signals indicate the position in the molecule. The formula shows the connectivity information as red bonds.

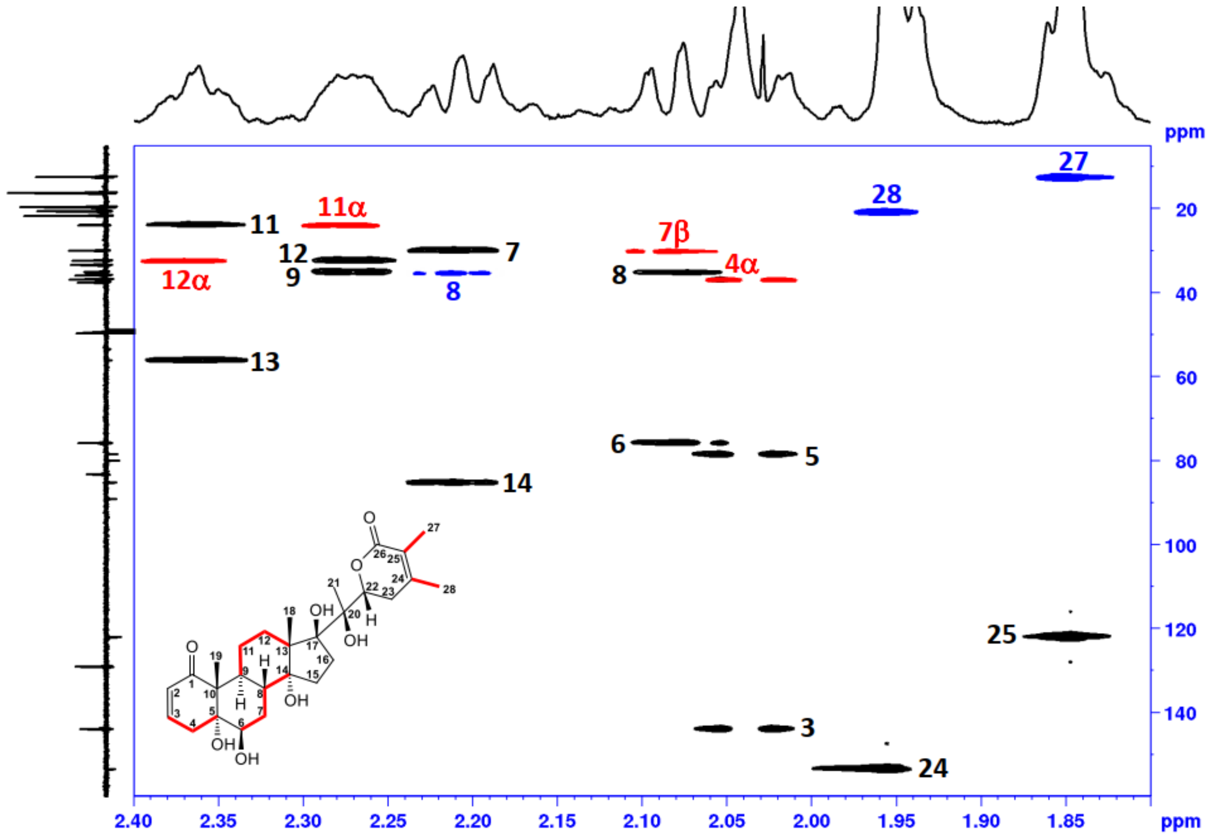

SI Figure 28: Detail of the phase edited and refocused 1,1-ADEQUATE spectrum of the metabolite withanolide S superimposed with the ^{1}H-^{13}C HSQC of the same spectral range (130 µg in 30 µl MeOH-d_3). Red and blue signals indicate $^{1}J_{CH}$ resonances, numbers next to signals indicate the position in the molecule. The formula shows the connectivity information as red bonds.

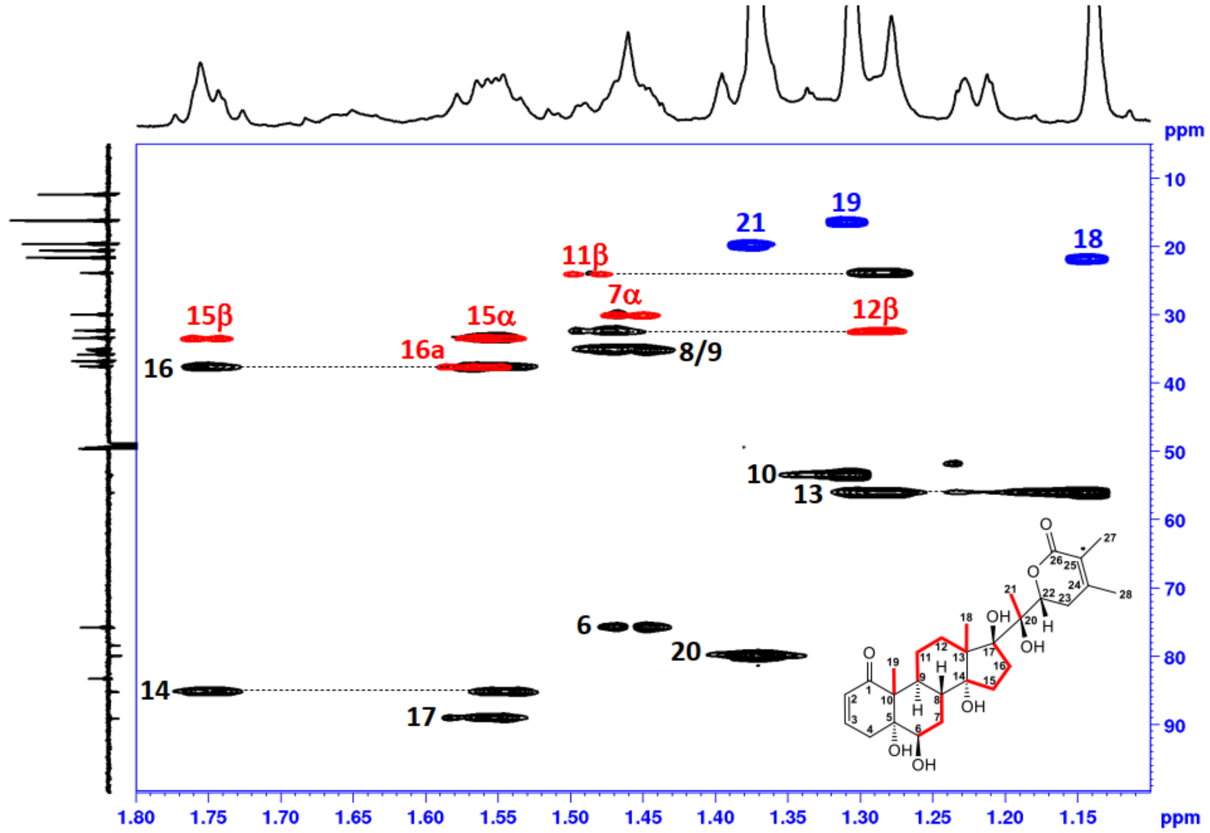

SI Figure 29: Detail of the phase edited and refocused 1,1-ADEQUATE spectrum of the metabolite withanolide S superimposed with the ^{1}H-^{13}C HSQC of the same spectral range (130 µg in 30 µl MeOH-d_3). Red and blue signals indicate $^{1}J_{CH}$ resonances, numbers next to signals indicate the position in the molecule. The formula shows the connectivity information as red bonds.

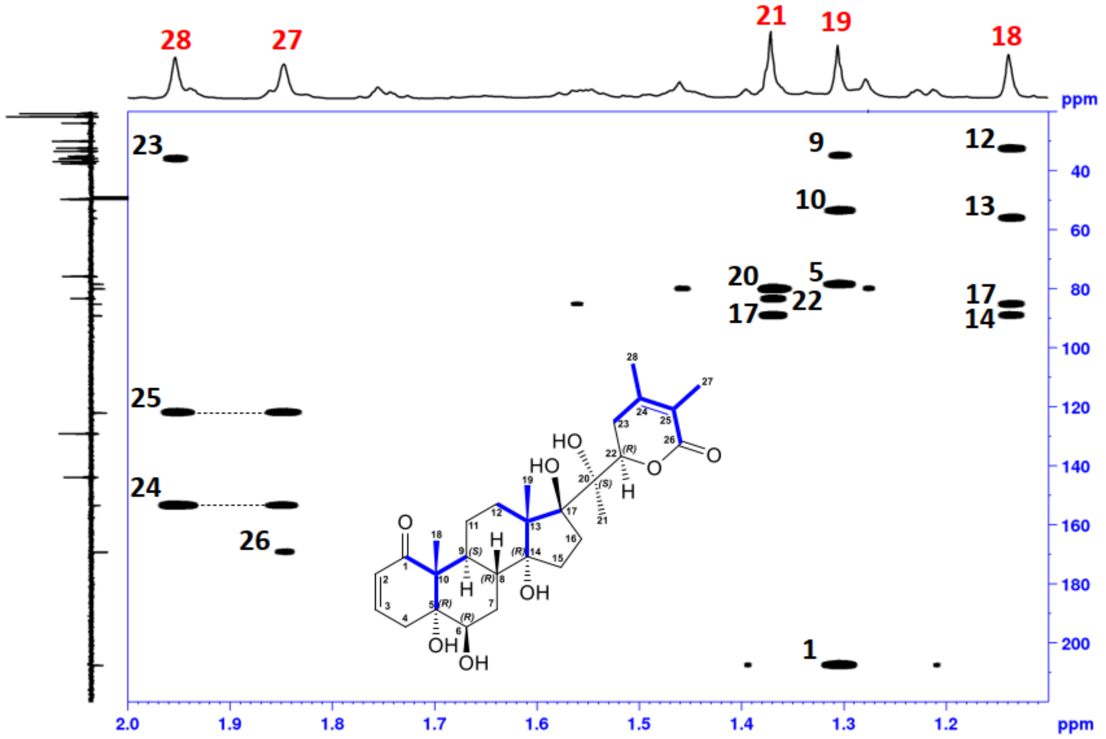

SI Figure 30: Detail of the ^{1}H-^{13}C HMBC spectrum of the metabolite withanolide S (130 µg in 30 µl MeOH-d_3). Red numbers on the $F_2(^{1}H)$ projection indicate the position of methyl groups in the molecule, black numbers next to the $^{2,3}J_{CH}$ correlations indicate the position in the molecule. Blue bonds in the formula show the extractable information.

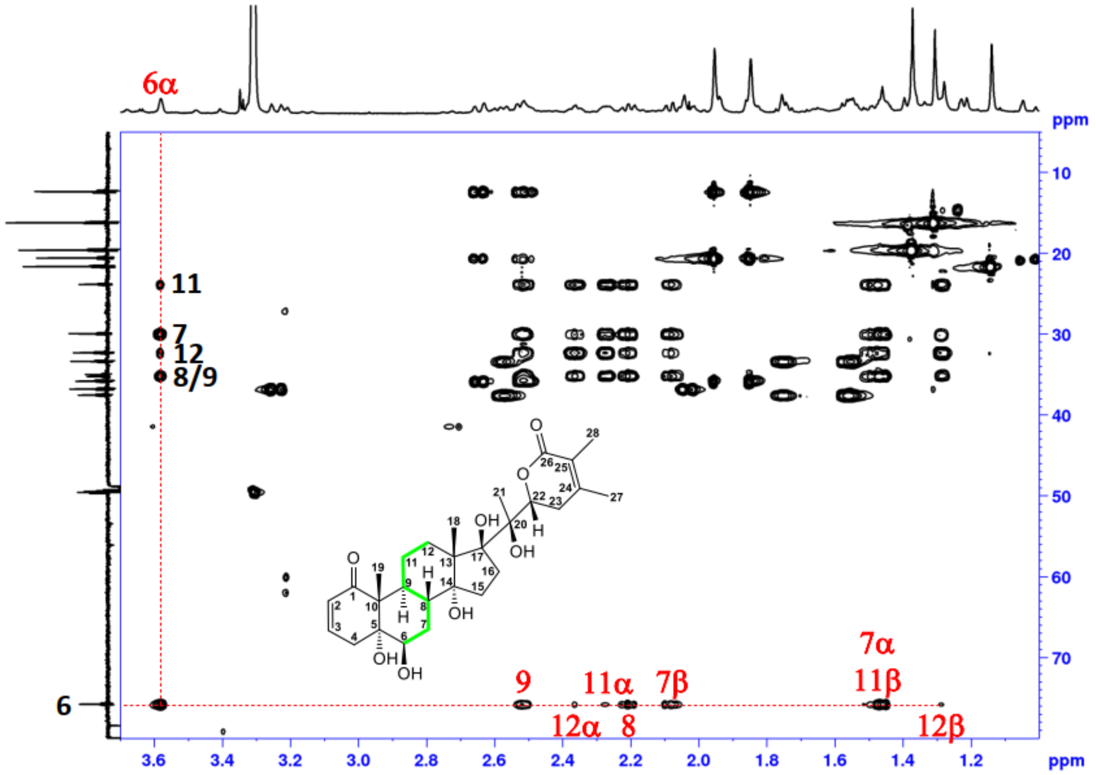

SI Figure 31: Detail of the ^{1}H-^{13}C HSQC-TOCSY spectrum of the metabolite withanolide S (130 µg in 30 µl MeOH-d_3). The red number on the F_2(^{1}H) projection indicates the position in the molecule, black numbers next to signals in the spectrum indicate the position in the molecule characterised by δ_C shifts. Red numbers indicate the position in the molecule characterized by δ_H shifts. The bonds highlighted in green in the formula show the spin system. Mixing time for the experiment was 120 ms.

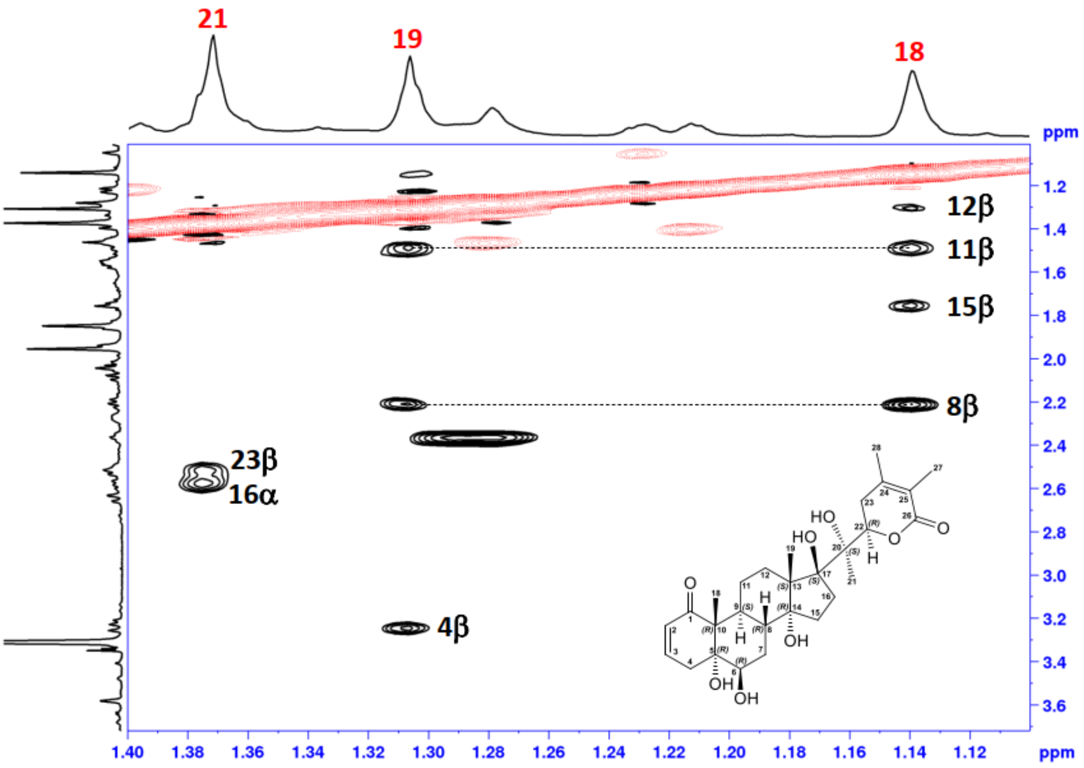

SI Figure 32: Detail of the ^1H-^1H ROESY spectrum of the metabolite withanolide S (130 µg in 30 µl MeOH-d_3). The red number on the F_2(^1H) projection indicates the position in the molecule, black numbers next to signals in the spectrum indicate the position in the molecule.

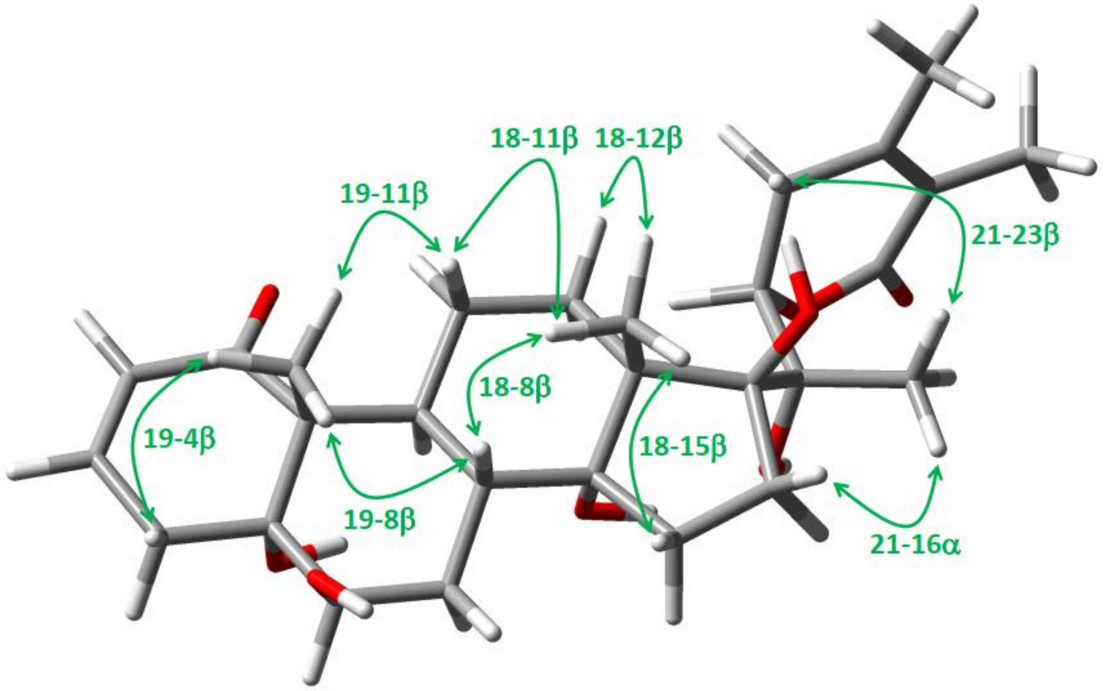

SI Figure 33: Graphical interpretation of the ROESY information extracted from **SI Figure 32**.

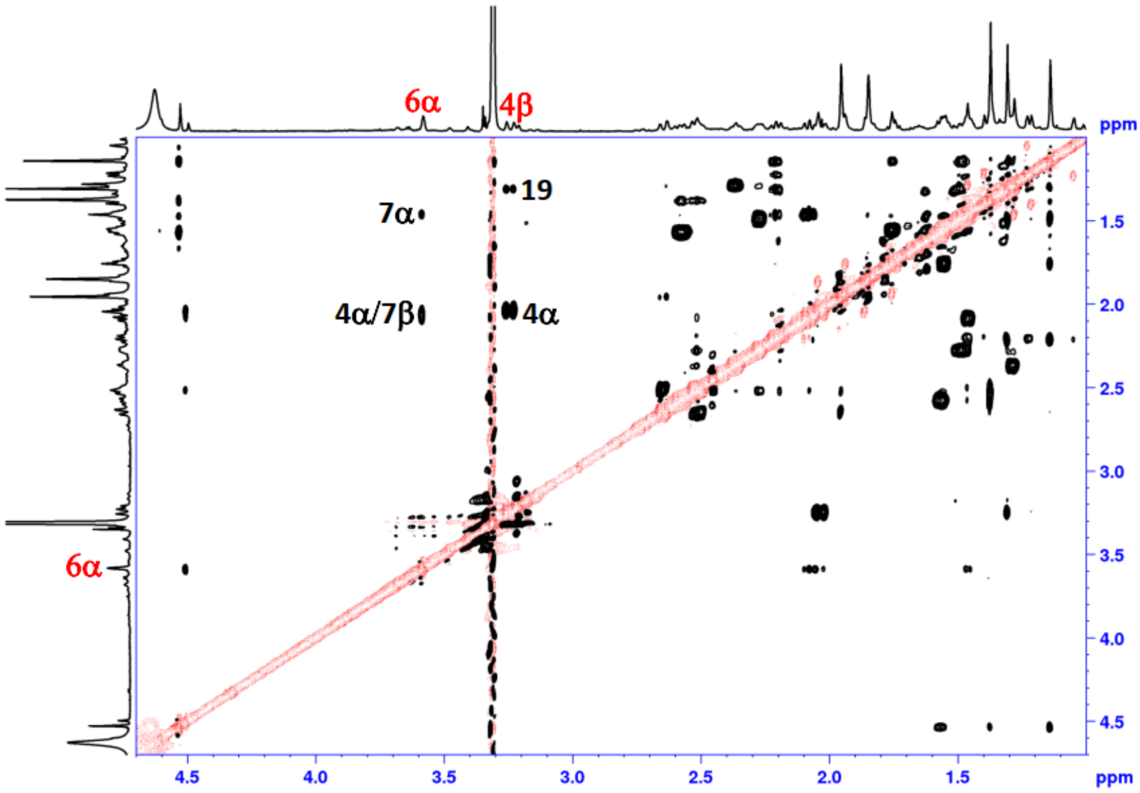

SI Figure 34: Detail of the ^1H-^1H ROESY spectrum of the metabolite withanolide S (130 µg in 30 µl MeOH-d_3). The red number on the F_2(^1H) projection indicates the position in the molecule, black numbers next to signals in the spectrum indicate the position in the molecule.

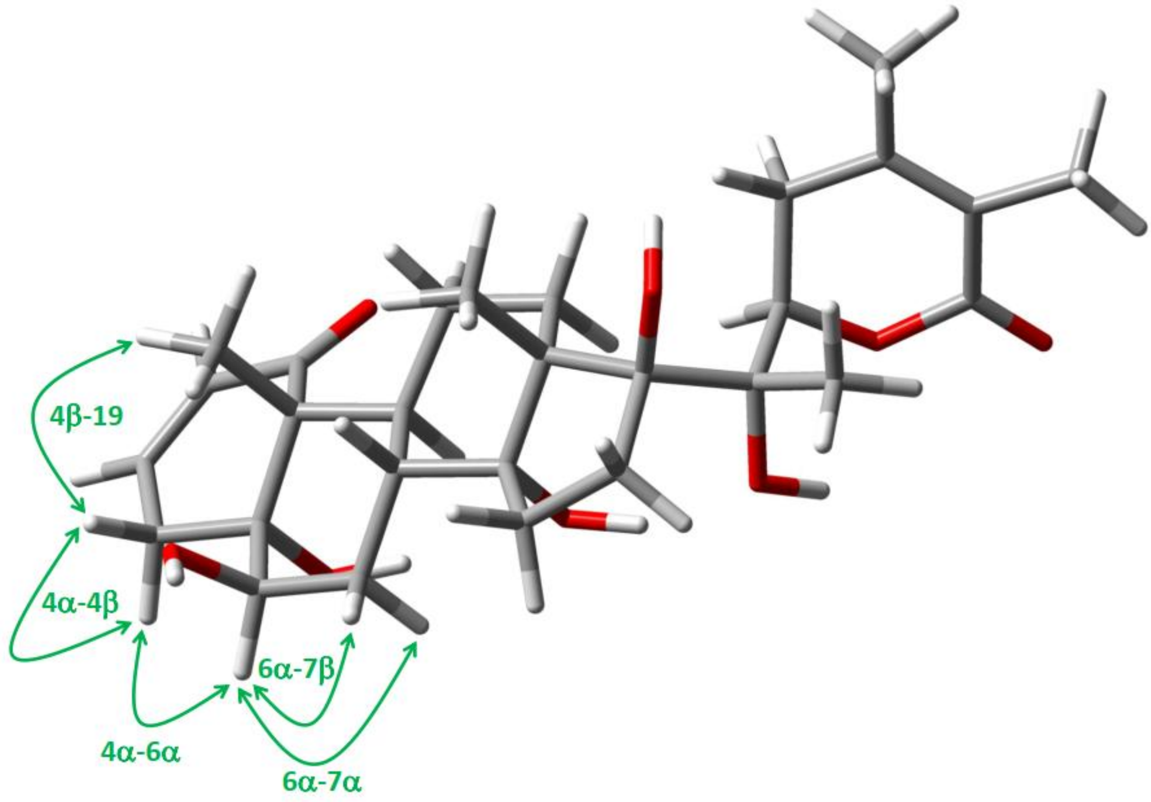

SI Figure 35: Graphical interpretation of the ROESY information extracted from **SI Figure 34**.

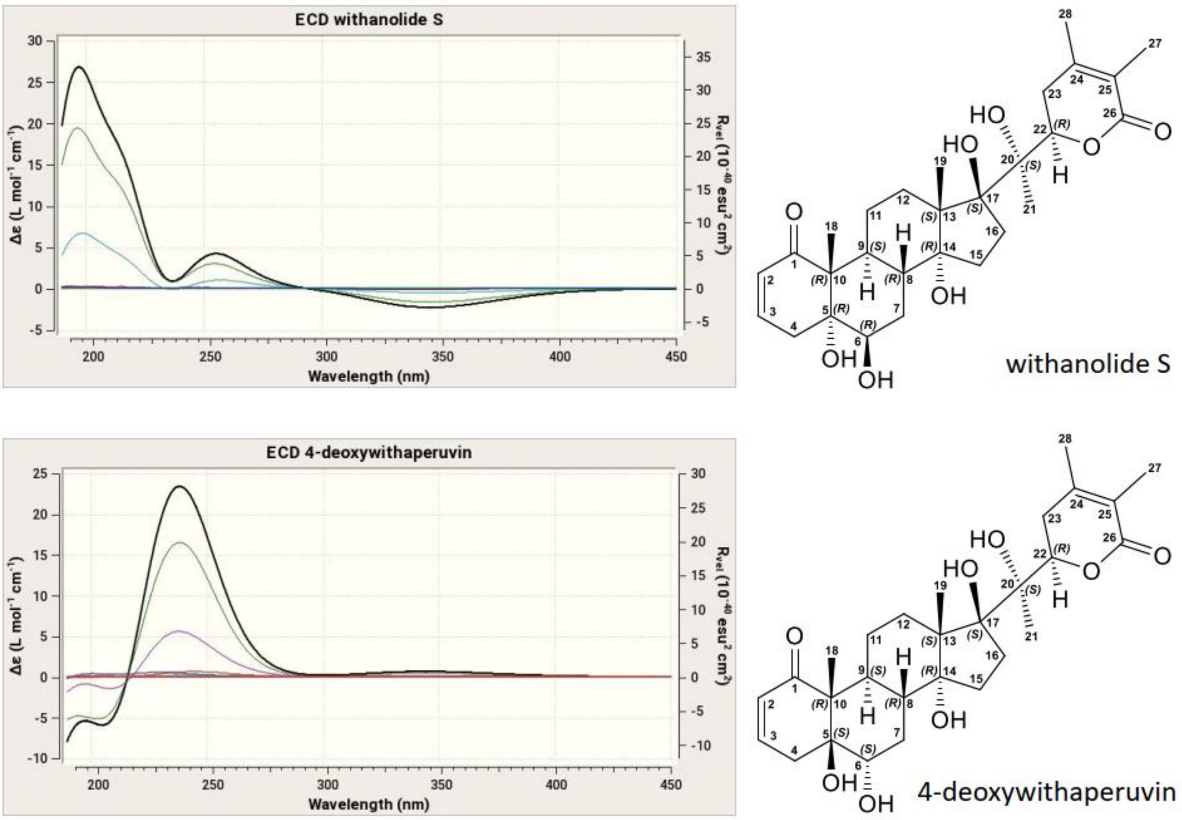

SI Figure 36: Calculated Electronic Circular Dichroism (ECD) spectra of withanolide S and 4-deoxywithaperuvin.

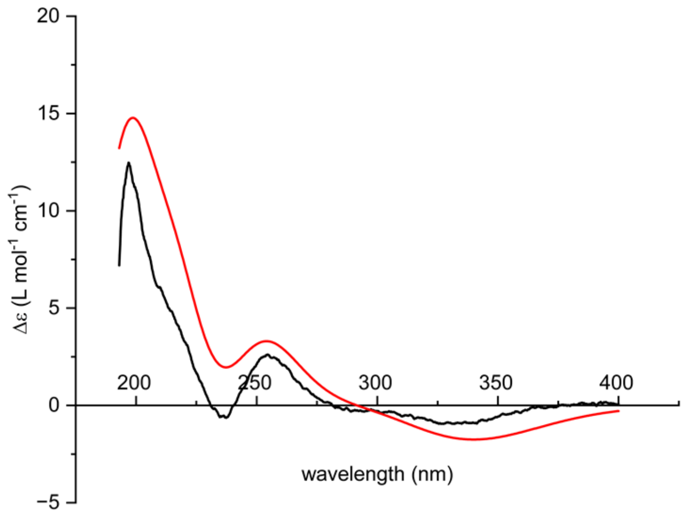

SI Figure 37: Experimental (black) and calculated (red) ECD spectra of withanolide S.

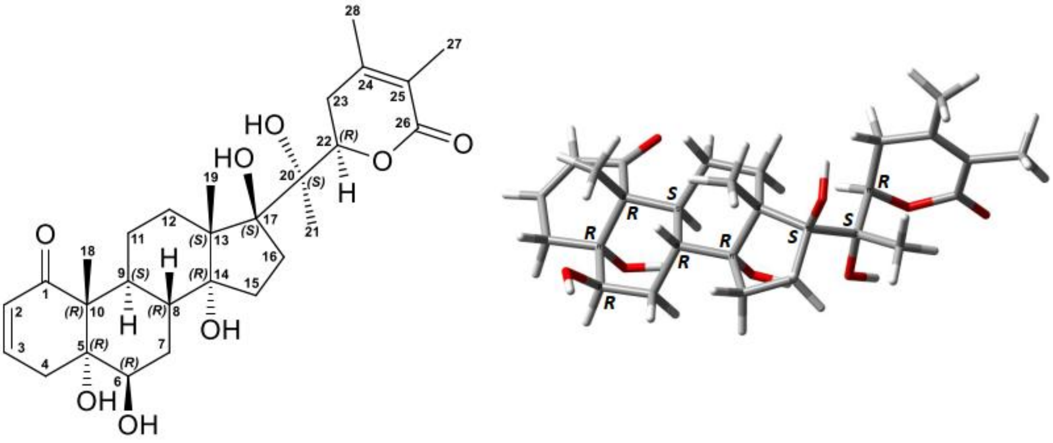

SI Figure 38: Planar structure and the molecular model of withanolide S with stereo descriptors. The positioning of the stereo structure corresponds to that of the X-ray result of physaperuvin G (Figure 2 from ref (25). The stereochemistry of C-5 and C-13 was incorrectly described as C5(S) and C13(R) respectively in the caption to Figure 2 of that publication.

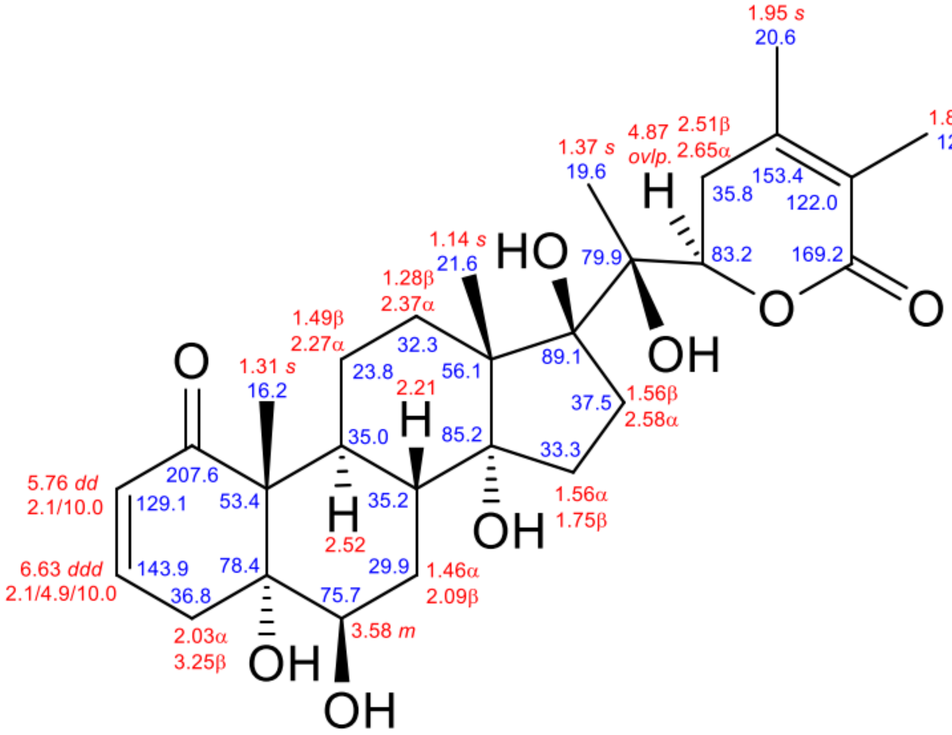

SI Figure 39: Structure of withanolide S isolated from feces of larvae after feeding [U-^{13}C]4β-hydroxywithanolide E). ^{1}H chemical shifts (δ ppm) and multiplicities together with the relative orientation towards the structural plane are given in red, ^{13}C chemical shifts (δ ppm) are given in blue.

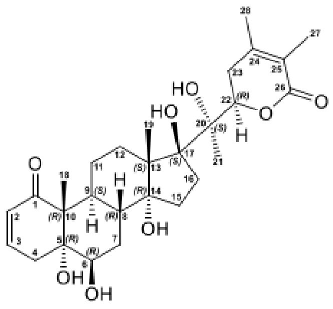

withanolide S

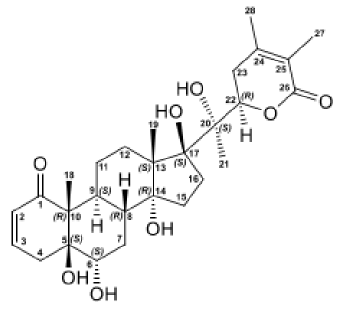

4-deoxywithaperuvin

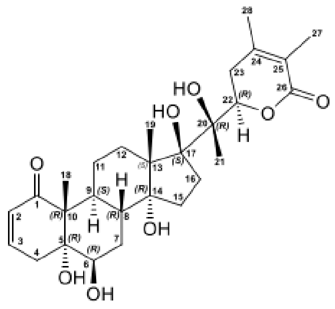

physaperuvin G

SI Figure 40: Possible metabolite structures ($C_{28}H_{40}O_8$) based on connectivity and oxygenation.

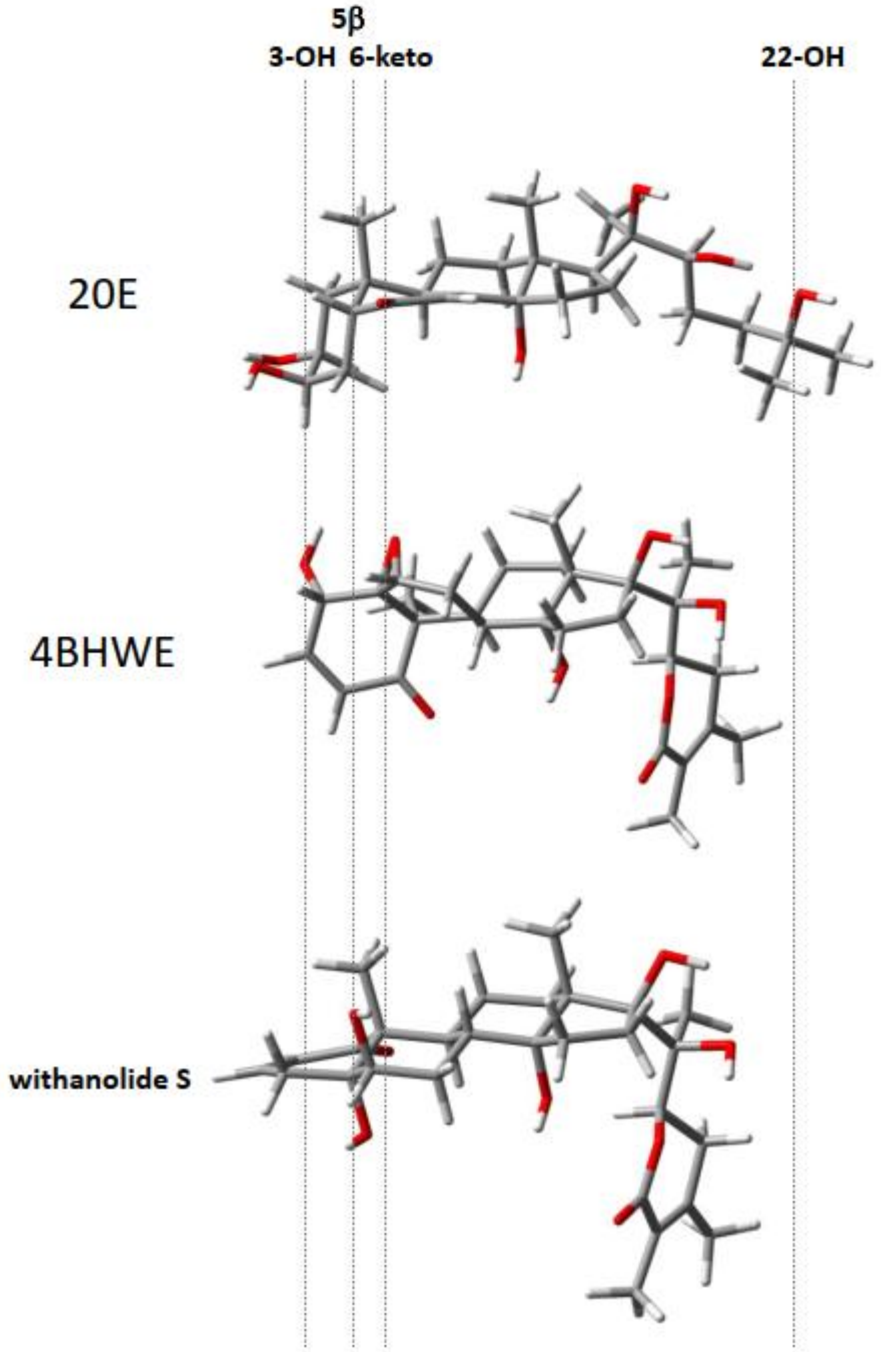

SI Figure 41: Molecular models of 20-hydroxyecdysone (20E), 4β-hydroxywithanolide E (4BHWE), and withanolide S. Structures were first optimized using a semi-empirical model (PM6), then analyzed regarding their conformers. The picture shows the lowest energy conformers. Grey dotted lines mark the structural features necessary for ecdysone activity.

SI Table 1: Chemical shift data of the metabolite withanolide S (δ_H/δ_C) and data reported for physaperuvin G (δ_H*/δ_C*) from ref (25).

pos.	δ_H	δ_H*	*mult.*, J_{HH} [Hz]	δ_C	δ_C*
**1**	**-**	*-*	-	**207.6**	*207.8*
**2**	**5.76**	*5.76*	*dd*, 2.1/10	**129.1**	*129.3*
**3**	**6.63**	*6.63*	*ddd*, 2.1/4.9/10.0	**143.9**	*144.0*
**4α**	**2.03**	*2.05*	*m*	**36.8**	*36.8*
**4β**	**3.25**	*3.22-3.25*	*m*	**36.8**	*36.8*
**5**	**-**	*-*	-	**78.4**	*78.3*
**6**	**3.58**	*3.58*	*m*	**75.7**	*75.8*
**7α**	**1.46**	*2.07-1.46*	*m*	**30.0**	*30.0*
**7β**	**2.09**	*2.07-1.46*	*m*	**30.0**	*30.0*
**8β**	**2.21**	*2.2*	*m*	**35.3**	*35.1*
**9α**	**2.52**	*2.56*	*m*	**35.0**	*35.3*
**10**	**-**	*-*	-	**53.4**	*53.5*
**11α**	**2.27**	*2.29-1.49*	*m*	**23.8**	*23.9*
**11β**	**1.49**	*2.29-1.49*	*m*	**23.8**	*23.9*
**12α**	**2.37**	*2.37-1.29*	*m*	**32.3**	*32.4*
**12β**	**1.28**	*2.37-1.29*	*m*	**32.3**	*32.4*
**13**	**-**	*-*	-	**56.1**	*56.1*
**14**	**-**	*-*	-	**85.2**	*85.1*
**15α**	**1.56**	*1.75-1.55*	m	**33.4**	*33.4*
**15β**	**1.75**	*1.75-1.55*	m	**33.4**	*33.4*
**16a**	**1.56**	*2.58-1.56*	m	**37.5**	*37.5*
**16b**	**2.58**	*2.58-1.56*	m	**37.5**	*37.5*
**17**	**-**	*-*	-	**89.1**	*89.1*
**18**	**1.14**	*1.14*	*s*	**21.6**	*21.7*
**19**	**1.31**	*1.31*	*s*	**16.2**	*16.2*
**20**	**-**	*-*	-	**79.9**	*79.9*
**21**	**1.37**	*1.38*	*s*	**19.6**	*19.6*
**22**	**4.87**	*4.87*	*m*	**83.3**	*83.3*
**23a**	**2.51**	*2.65*	*m*	**35.8**	*35.9*
**23b**	**2.65**	*2.65*	*m*	**35.8**	*35.9*
**24**	**-**	*-*	-	**153.4**	*153.4*
**25**	**-**	*-*	-	**122.0**	*122.1*
**26**	-	*-*	-	**169.2**	*169.2*
**27**	1.85	*1.85*	*s*	**12.4**	*12.4*
**28**	1.95	*1.96*	*s*	**20.6**	*20.6*
